# Biodosimetry, can it find its way to the nuclear medicine clinic?

**DOI:** 10.3389/fnume.2023.1209823

**Published:** 2023-07-25

**Authors:** Julie Bolcaen, Nastassja Combrink, Kaat Spoormans, Stuart More, Charlot Vandevoorde, Randall Fisher, Janke Kleynhans

**Affiliations:** ^1^ Radiation Biophysics Division, SSC Laboratory, iThemba Laboratory for Accelerator Based Sciences (iThemba LABS), Cape Town, South Africa; ^2^ Nuclear Medicine Division, Faculty of Medicine and Health Sciences, Stellenbosch University, Cape Town, South Africa; ^3^ Nuclear Medicine and Molecular Imaging, Department of Imaging and Pathology, University of Leuven, Leuven, Belgium; ^4^ Division of Nuclear Medicine, Department of Radiation Medicine, University of Cape Town, Cape Town, South Africa; ^5^ Biophysics Departement, GSI Helmholtzzentrum für Schwerionenforschung GmbH, Darmstadt, Germany; ^6^ Radiopharmaceutical Research, Department of Pharmacy and Pharmacology, Catholic University of Leuven, Leuven, Belgium

**Keywords:** [^177^Lu]Lu-PSMA-RLT, [^177^Lu]Lu-DOTA-TATE/TOC, Iodine-131, dicentric chromosome assay (DCA), γH2AX foci assay, cytokinesis-block micronucleus assay (CBMN), gene transcript analysis, biodosimetry and nuclear medicine

## Abstract

Personalised dosimetry based on molecular imaging is a field that has grown exponentially in the last decade due to the increasing success of Radioligand Therapy (RLT). Despite advances in imaging-based 3D dose estimation, the administered dose of a therapeutic radiopharmaceutical for RLT is often non-personalised, with standardised dose regimens administered every 4–6 weeks. Biodosimetry markers, such as chromosomal aberrations, could be used alongside image-based dosimetry as a tool for individualised dose estimation to further understand normal tissue toxicity and refine the administered dose. In this review we give an overview of biodosimetry markers that are used for blood dose estimation, followed by an overview of their current results when applied in RLT patients. Finally, an in-depth discussion will provide a perspective on the potential for the use of biodosimetry in the nuclear medicine clinic.

## 1. Introduction

Recently, the number of patients treated with radioligand therapy (RLT) has increased drastically (1, 2). RLT has been recognized as an effective targeted approach to treat many types of malignancies, often at late stages when conventional therapy has proven ineffective. The common challenge with cytotoxic treatments using ionising radiation is to deliver the highest possible dose of radiation to the target to achieve tumour control, while minimising excessive normal tissue toxicity (3, 4). Imaging-based dosimetry allows the quantification of the absorbed dose (i.e., radiation energy deposited per unit mass) to a certain target (i.e., receptor expressing cancer) or organ, based on activity distributions from single photon emission computed tomography/computed tomography (SPECT/CT) and scintigraphy images (5). Imaging-based dosimetry as applied in RLT follows the principle of Medical Internal Radiation Dosimetry (MIRD), which provides a standardised framework and methodology for the calculation of the absorbed dose delivered by the radiopharmaceuticals to both the tumour and the organs at risk. However, the current practice in the nuclear medicine clinic today is to administer a fixed amount of radioactivity to the patient, expressed in Becquerel (Bq) or Curie (Ci) of radioactivity, instead of an absorbed dose as is the case in external beam radiation therapy (EBRT) (5).

**Figure 1:**
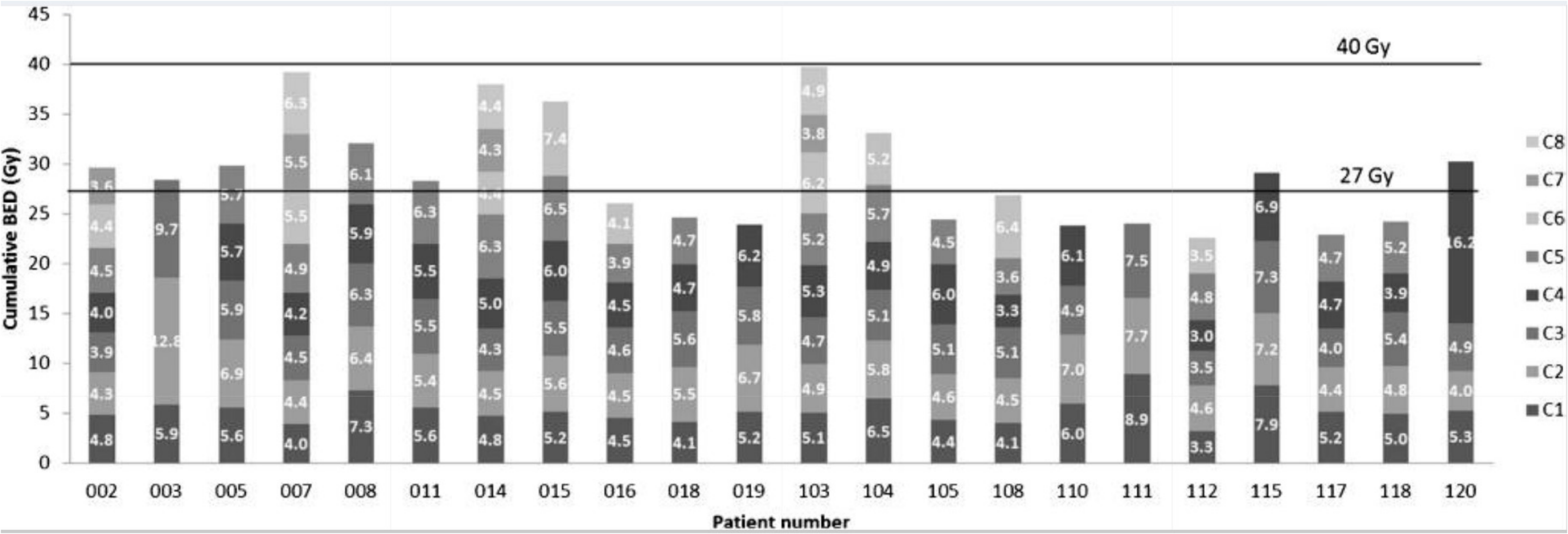
The cumulative biologically effective dose (BED) per cycle of individual patients and the personalised number of cycles received (ranging from 3 to 8 cycles) per patient (C1-C8) (reprinted with permission from Sundlöv et al., 2017 ^©^Springer, 2017) (19).

Overall, the adoption of a MIRD-based personalised dosimetry approach during RLT would most likely result in higher therapy efficacy and a reduction of side effects. The incidences of the most common early and late-stage toxicities of concern in RLT are provided in Table 1. The ideal therapeutic-to-toxicity ratio may be achieved by adjusting the dosing of each patient to their unique pharmacokinetics and dosimetry. Even more ideal would be to consider the intrinsic radiosensitivity of the patient’s healthy or diseased tissue (18). An elegant application of this approach was reported during a phase II trial performed with [^177^Lu]Lu-DOTA-TATE. The radioactivity administered was adjusted according to the calculated radiation dose received by the kidneys through the application of MIRD (19). These patients received a maximal cumulative kidney dose of up to 27 ± 2 Gy, but more importantly, in patients without risk, this maximum dose could be increased to 40 ± 2 Gy. The patients selected for the higher dose group had no risk factors for haematological or renal toxicity, non-progressive disease, or good tolerance. These limits were based considering previous experiences with EBRT. The findings of this study clearly demonstrated that individualising treatment was important, as a considerable variety of amounts of radioactivity had to be administered to patients based on the MIRD calculations. Furthermore, the received radiation dose calculated to be delivered to the kidney was significantly different between patients and between the treatment cycles in each individual patient. The number of cycles that could be safely administered per patient was also subject to high intra-patient variability (Figure 1). This study confirmed the role MIRD could play in personalising the administered radiopharmaceutical activity. However, dosimetry-based personalised dosimetry needs further development since current data is based on dose-response relationships for tumour tissue and normal tissues extrapolated from EBRT and the linear-quadratic model (LQ) or the calculation of radiobiological effects (19). It is well established that the extrapolation of EBRT to RLT is not straightforward. In contrast to EBRT, RLT is characterised by an inherent heterogeneous and mixed radiation field, with protracted exposure times, and a considerably lower absorbed dose rate (20).

**Table 1:** Incidence of early and late-stage toxicities experienced with current RLT.

Radiopharmaceutical	Early-stage toxicity	Late-stage toxicity	Ref
[^177^Lu]Lu-PSMA RLT	Grade 3–4 hematotoxicity (<10%). Grade 3–4 xerostomia (<5%)—transient. Grade 1–2 fatigue (34.7%)	None reported to date.	(6) (7)
[^225^Ac]Ac-PSMA RLT	Grade 3–4 hematotoxicity (33%). 23% of patients stop therapy due to xerostomia	Delayed nephrotoxicity reported in case studies.	(8) (9) (10)
Beta-emitter based Somatostatin targeting PRRT*	Severe (grades 3 and 4), reversible bone marrow toxicity <10%–13% of cycles with [^90^Y]Y-DOTA-TOC, 2%–3% of cycles with [^177^Lu]Lu-DOTA-TATE. These resolve in 8 weeks after therapy.	[^90^Y]Y-DOTA-TOC grade 4 and 5 kidney toxicity in 9.2% of patients. Long-term renal toxicity of grade 3–4 is less than 2% with [^177^Lu]Lu-DOTA-TATE. Sporadic cases of myelodysplastic syndrome or acute myelogenous leukaemia have been reported in 2–3% of patients at a median of 2 years post-therapy.	(11) (12)
Radium-223 dichloride	Grade 3–4 thrombocytopenia and neutropenia (≥10%)		(13)
High dose radioiodine	Transient decrease in leukocytes and platelets may occur 6–10 weeks post therapy. The incidence is dependent on the administered activity. Radiation thyroiditis (10–20%)	Reduced salivary gland function (24,9% after 1 year), Recurrent conjunctivitis (22.7%). Moderate decrease in leukocytes (4%). Although uncommon, the development of other malignancies has been described (<1%). Fertility issues are described, dose limit not yet established.	(14) (15) (16)
[^131^I]MIBG	Temporary myelosuppression lasting 4–6 weeks (<60%)	Rarely persistent haematological effects and induction of leukaemia have been reported.	(17)

(PRRT) Peptide receptor radionuclide therapy; (MIBG) meta-iodobenzylguanidine.

An alternative radiobiology-based dosimetry method is biodosimetry, which allows an individual dose estimation based on biological endpoints that are altered by ionising radiation exposure, or so-called radiation biomarkers (21). MIRD converts the accumulated amount of radioactivity in different tissues to a predicted radiation dose, considering individual differences in pharmacokinetics. Biodosimetry could improve this strategy by also considering the intrinsic radiosensitivity of the patient. As noted by O’Neil and Cornelissen (22), radiobiology-based biomarkers could be used, alongside dosimetry, to further understand normal tissue toxicity to refine the as high as safely administrable (AHASA) thresholds for each RLT agent, as well as to determine the as low as reasonably achievable (ALARA) minimum dose thresholds that are necessary to achieve a satisfactory therapeutic outcome. A dose-response relationship could be established between the radiation dose and the radiobiological response for each tissue/individual patient (22).

Several radiation biomarkers have been studied up until now to assess the effects of therapeutic radionuclides (5). This review includes an overview of the different biodosimetry assays and a summary of their results in clinical studies correlating image-based dosimetry with biodosimetry. Finally, an in-depth discussion will give a perspective on the utility of biodosimetry markers in the nuclear medicine clinic.

## 2. Current clinical practice of RLT dosimetry and its shortcomings

In conventional EBRT, a clear dose-response relationship has been demonstrated for both tumour response and toxicity response (22). For RLT, a similar radiation dose-response relationship is expected, with the focus not on the administered activity (Bq) but rather on the absorbed dose, i.e., radiation energy deposited per unit mass of tissue (Gy) (2, 23). However, the relationship between administered activity and absorbed dose remains to be established. In conventional RLT dosimetry methods, the absorbed dose to tumour and healthy tissue is estimated based on activity distribution, evaluated at either pre- or post-treatment time points (24–26). This information, together with dose-response models, could be used to modulate the administered activity to either limit the toxicity to healthy tissue or maximise the dose to the tumour (24).

Image-based dosimetry in RLT follows the principle of MIRD (27). Here, a time-integrated activity coefficient (TIAC) is multiplied by a so-called S-value. The latter value is the absorbed dose per nuclear decay, which depends largely on the characteristics of the radionuclide and the anatomy of the patient. For multiple radionuclides and realistic patient phantoms, S-values for whole organs and spherical tumour volumes are tabulated and can be accessed via software tools, such as OLINDA, MIRDOSE, IDAC-DOSE and MIRDcalc (28). The TIAC requires integration of the activity distribution over time and thus sampling of the activity distribution at multiple time points. Quantitative nuclear scans can provide this activity distribution either before treatment with a diagnostic surrogate radionuclide or after treatment via co-emitted gamma-photons of the therapeutic radionuclide (29, 30).

An example of pre-treatment dosimetry is during therapy for benign thyroid disease with iodine-131. It is advised that an individualised amount of radioactivity be administered based on pre-treatment dosimetry calculations performed on radioiodine uptake measures (with diagnostic iodine-123) (31). Some recent studies retrospectively investigated gallium-68 diagnostic radiopharmaceuticals for pre-therapy treatment planning of lutetium-177 therapy, but this is not standard practice (32, 33). Pre-treatment dosimetry assumes that the diagnostic surrogate is chemically identical to the therapeutic one, that the uptake is linear with administered activity, and that the patient’s condition does not change over time (28). However, the final biokinetics of the therapeutic drug might be considerably different compared to the diagnostic surrogates, resulting in poor dose estimation (34). Post-treatment dosimetry overcomes this issue but requires the co-emission of gamma-photons suitable for imaging. However, not all therapeutic radionuclides have theranostic potential. The post-treatment dose estimations provide valid information for activity modulation in upcoming treatment cycles, but also for the establishment and further optimisation of robust dose-response relationships. As an example, [^177^Lu]Lu-PSMA-RLT dosimetry offers the possibility of post-treatment dosimetry. However, the current European Association of Nuclear Medicine (EANM) guidelines propose a “one-size-fits-all” routinely administered radioactivity of 7.4 GBq (every 6 weeks for four to six cycles) for [^177^Lu]Lu-PSMA-RLT and this approach is rarely followed (6, 35). The fixed dose of 7.4 GBq of [^177^Lu]Lu-PSMA-RLT or [^177^Lu]Lu-DOTA-TATE is based on the accumulation of single-institution trials and real-world clinical experiences and was not studied in prospective dose escalation studies. However, these fixed-doses were evaluated in the notable clinical trials (VISION and NETTER-1) as reasonably safe and effective in the majority of patients and are therefore widely implemented and accepted in nuclear medicine practices. This led to a “one size fits all approach” and unfortunately, this excludes the mandate of personalised medicine. However, it is well reported that a more personalised balance between administered radioactivity and toxicity provides better patient outcomes than fixed doses (36).

For solid organs or cancerous structures (e.g., kidney, liver, solid tumours), image-based dosimetry results in dose estimations with an error margin of about 10%–15% (28). However, for small metastases or diffused organs, such as the radiosensitive bone marrow, this image-based dosimetry is even less accurate. For bone marrow specifically, the activity in blood samples is measured and used to estimate bone marrow dose. Furthermore, image-based dosimetry results in whole organ-absorbed dose estimations but neglects suborganic and definitly subcellular dose heterogeneities, which becomes more important for radionuclides with a short emission range, such as alpha-particle and Auger electron emitting radionuclides (40).

An alternative approach for personalised dosimetry could be the use of micro-sized thermoluminescent dosimeters (TLD). This has been applied with some success to EBRT and brachytherapy, where the detectors are placed on the skin, in body activities or behind the patient (41–43). Since there is limited research being done on their applicability in RLT, it is assumed that TLDs will have to be implanted into the tumour tissue to have accurate measurements. Depending on the advancement in technology, the current state of the application would require a pre-therapy and post-therapy operation or application into the tumour. It is envisioned to only provide dosimetry coverage for one or two tumour sites per patient, as application to all micro-metastases as well as critical organs seems too invasive. Another shortcoming might be the small volume of recording of the TLD, which is limited by the range and location of implantation. This could have substantial implications for alpha-particle emitters and auger emitters due to their small penetration range. Since the TLD’s will receive radiation over a prolonged period of time due to the residence of the radiopharmaceutical in the tissue, this is in contrast with the short exposure of minutes with ERBT. The TLD must therefore be made physiologically acceptable so as to interact locally with the tissue, be broken down biologically, and still accurately provide measurements after being removed. This application is discussed in more depth by Yorke and co-workers (44).

**Figure 2:**
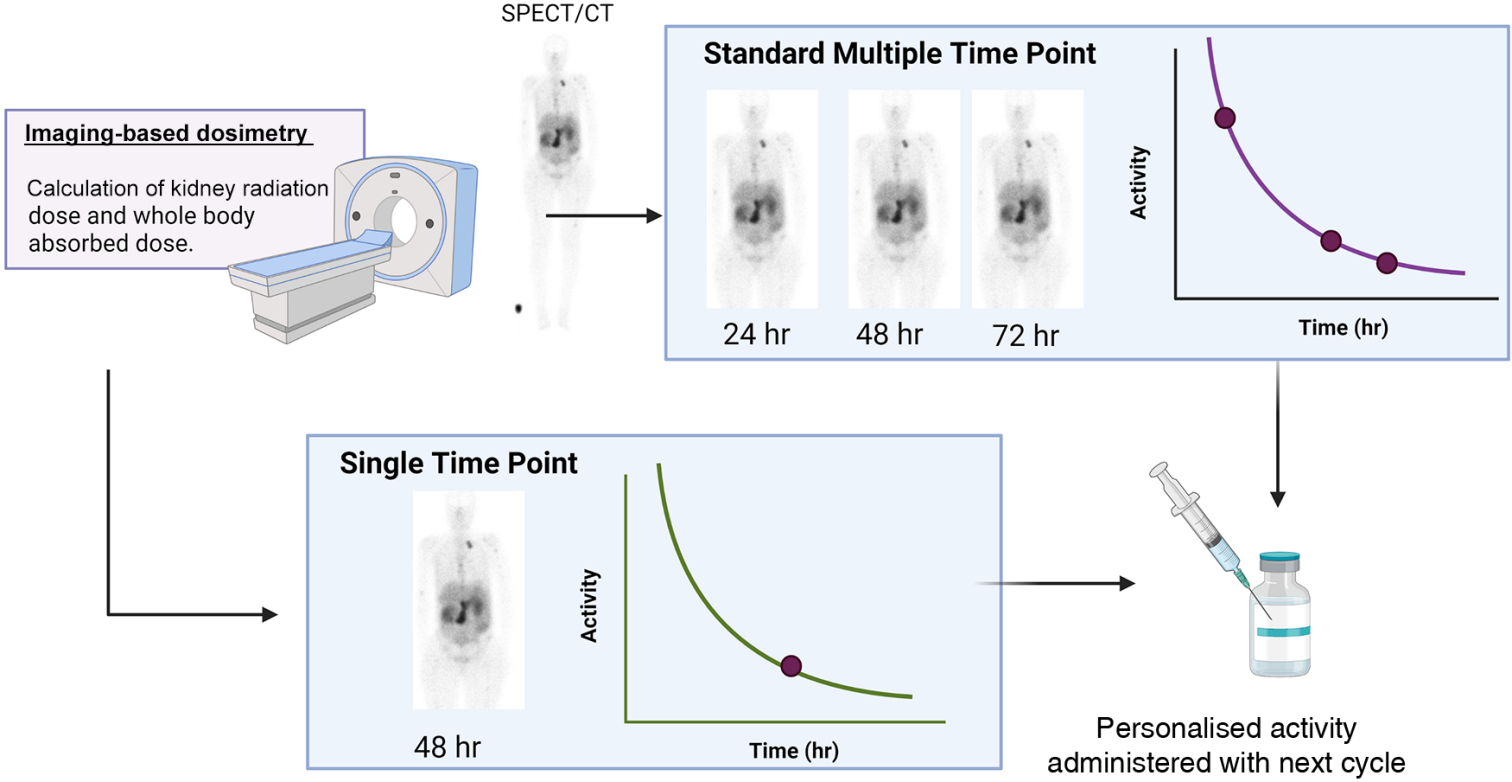
A schematic of the difference between multiple time point dosimetry and single time point dosimetry (created with Biorender.com).

One of the major shortcomings of dosimetry-based planning is the inconvenience for the patient and the constraints on the resources of the clinic. The contribution of this factor towards the inefficient implementation of MIRD-based adjustments to patient treatment should not be underestimated. Currently, a concerted effort is being made towards the introduction of single-time-point dosimetry (STPD) (Figure 2) in many institutions for application in [^177^Lu]Lu-SSTR2 and iodine-131 therapy. As the name suggests, in STPD, the activity distribution is only measured at a single time point, and assumptions are made for the pharmacokinetics at earlier and later times. The assumption of a mono-exponential decay combined with a population mean effective decay time resulted in acceptable dose errors (<30%) for [^177^Lu]Lu-DOTATATE and kidney dosimetry, while it was less promising for [^177^Lu]Lu-PSMA and bone marrow and tumour dosimetry (45–48). The population mean effective decay is obtained from known biokinetics from average population distribution data. In such a case, a pharmacokinetic model is created from the general population, into which individual scan data can be fed to predict the dose for the individual patient. It is important to note that there is still disagreement in the literature regarding the validity of this approach (28, 37–39). Nonetheless, multiple imaging methods require both advanced equipment and specialised staff for image processing and analysis, which are currently not widely available. Secondly, it is important to note that, as with MIRD in general, STPD still does not solve the problem associated with current data models being based on dose-response relationships for tumour tissue and normal tissues extrapolated from EBRT and the linear-quadratic model or the calculation of radiobiological effects.

The current clinical practice of RLT dosimetry is greatly varied across different treatment facilities and treatments. However, in general, the use of pre-treatment dosimetry is limited to patient screening and post-treatment dosimetry is used to gather retrospective information. To allow better dosimetry integration into routine clinical practice, more robust dose-response data are required. This necessitates the performance of multicentre clinical trials with a streamlined and standardised dosimetry approach. Furthermore, it is not enough that extrapolations are made from ERBT models. This necessitates multicentre clinical trials with a streamlined and standardised dosimetry approach.

## 3. Available biodosimetry options to support MIRD and blood dose estimations

Biodosimetry comprises the detection of biological alterations induced by exposure to ionising radiation, which are used to quantify and estimate the individual absorbed radiation dose (49). Deoxyribonucleic acid (DNA) is recognised as one of the principal targets for the biological effects of ionising radiation, particularly the induction of double-strand breaks (DSBs) and single-strand breaks (SSBs). While most radiation-induced lesions are repaired successfully by the cell’s DNA damage response pathways, DSBs are the most difficult lesions to accurately repair, and misrepair can lead to the formation of chromosome-type aberrations (20, 50). Insertions and translocations are classified as stable aberrations which persist over time, whereas dicentrics, rings, and acentric fragments are unstable aberrations that will disappear over time during cell division (22, 51).

In the context of RLT, biodosimetry could complement MIRD and allow the estimation of the absorbed radiation dose that has been delivered to the blood as a surrogate for the haematopoietic system of an individual patient during therapy (52, 53). The calculated biological dose is not a measure for the whole-body dose but represents the dose to the blood volume and vascular walls, which can then be used as a surrogate marker for the absorbed dose to the bone marrow, which is the most radiosensitive tissue in the body (53–55). In general, a dose limit of 2 Gy to the bone marrow is considered acceptable (54). Table 1 indicates that most therapies suffer from bone marrow suppression, which is often presented as an early-onset toxicity in RLT patients. Furthermore, for many RLTs, there are still large uncertainties on the prediction of long-term haematological toxicity, including myeloid dysplasia and acute myeloid leukaemia, for which there are no known bone marrow dose-response relationships (19, 52, 56–58). The large error margin in MIRD calculations (often exceeding 10%–15%) makes this a less than accurate solution for the prediction of bone marrow toxicity (28, 40). Hence, the potential of biodosimetry to predict bone marrow toxicity is worth investigating. The latter could allow adjustments to the prescribed treatment (e.g., dose, timing) on an individual patient-by-patient basis.

Another crucial proposed application for biodosimetry is the measurement of dose-dependent DSBs in the renal cortex as a biological indicator for long-term radiation effects on the kidney. As previously indicated (Table 1), nephrotoxicity is often a concern during RLT. However, it is possible that a correlation between DSBs in peripheral blood lymphocytes (PBLs) and renal cortex tissue can be established (59). This would allow the accurate prediction of long-term nephrotoxicity and could allow adjustments to subsequent treatment cycles. This investigation was reported in a preclinical model, and translation to the clinic might be too risky as sampling kidney tissue from patients is invasive. For this application, both the *γ*-H2AX foci and gene transcript analysis have been investigated preclinically (59, 60).

For cytogenetic biodosimetry purposes, the circulating PBLs of patients suspected to be radiation exposed are most commonly used for a range of quantitative assays, including the dicentric chromosome assay (DCA), cytokinesis-block micronucleus (CBMN) assay, and FISH (Fluorescence in situ hybridization) methods. In this regard, PBLs are the ideal candidates for biological dosimetry because they are obtained by minimally invasive methods and sufficient cell numbers are extracted from the peripheral blood of the patients (1 ml of blood can contain up to 3 × 10^6^ lymphocytes in healthy individuals). Furthermore, T-lymphocytes are long-living circulating cells that can reach all body areas and can be considered “circulating dosimeters”. In addition, PBLs do not often undergo mitosis and retain the signs of radiation damage for longer periods of time. Also, there is a well-established, direct correlation of chromosomal aberrations with the amount of absorbed radiation dose (61, 62). The scoring of unstable chromosome-type aberrations in PBLs using the DCA remains the “gold standard” in biodosimetry due to its high specificity for irradiation exposure as well as the low background levels, making it one of the most reliable biological indicators of radiation exposure (63). Alternative cytogenetic assays have been commonly used over the past decades to assess absorbed radiation doses, including CBMN, premature chromosome condensation (PCC), and the scoring of fragments/rings (63).

More recently, the gamma-H2AX foci assay has been introduced in the biodosimetry landscape, owning to its high sensitivity (down to the mGy range) coupled with the added advantage that it does not require the induction of mitosis in PBLs, which reduces the assay turn-around time beyond the traditional cytogenetic assays (64). Another time- and high-throughput alternative biodosimetry assay that is growing in popularity is gene expression analysis (65). A brief discussion of these methods is presented here, along with pilot studies reported in the literature on their use in RLT.

### 3.1. Dicentric chromosome assay

**Figure 3:**
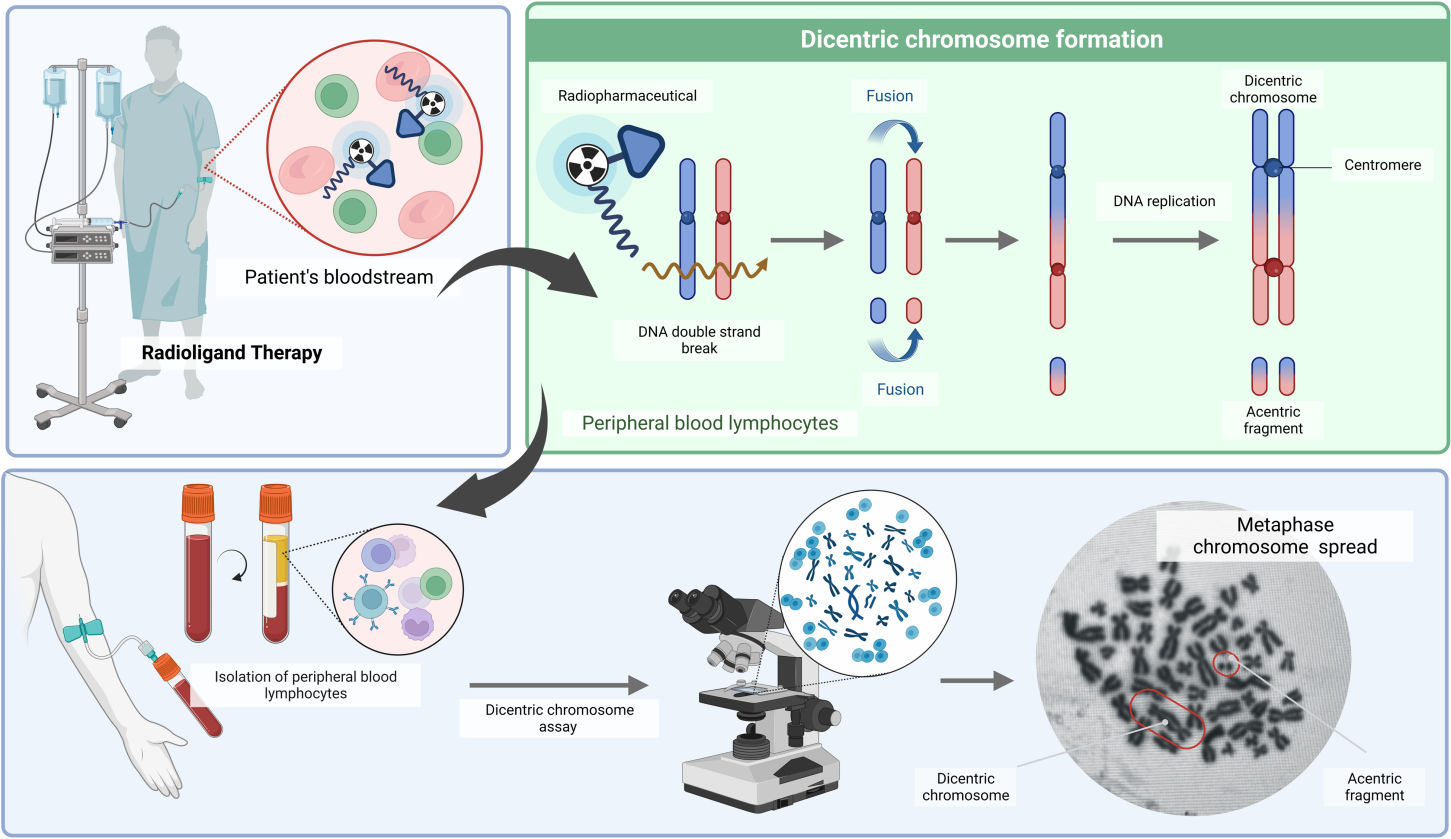
A schematic overview of the dicentric chromosome assay (DCA) (created with BioRender.com).

During the repair of DSBs, misrepair of broken chromosomes and abnormal chromosome replication can lead to the formation of a dicentric chromosome (DC) characterised by the presence of two centromeres. As a consequence of the formation of a DC, an acentric fragment will appear (Figure 3) (66). When these cells progress through mitosis, chromosomal aberrations will be recognised by the cell as abnormal, leading in either cell death or gives rise to inherited mutations (20, 55). Owing to its precision and sensitivity down to 0.1 Gy, DC is a widely used biodosimetry method that is medically and legally recognised (67). Complex chromosome-type aberrations are useful biomarkers to distinguish low and high linear energy transfer (LET) radiation qualities, and their identification could provide an optional approach for estimating the doses from mixed exposures (68).

In the field of nuclear medicine, investigations on chromosome-type aberration analysis in PBLs remain limited to iodine-131-based therapies (Table 2) (69–75). Some applications have also been reported in locally administered radionuclide therapies such as synovectomy treatment (76, 77). However, it is not so clear if local therapies would benefit from routine biodosimetry, and hence, this is not extensively covered in this review.

**Table 2:** Overview of clinical studies performing biodosimetry in RLT patients using chromosomal aberration analysis.

Patient population	Radio pharmaceutical	Timing post-administration	Main result	Reference
DCA assay				
Neuroblastoma (*n* = 1)	[^131^I]MIBG	Before treatment and 7 days after each cycle	Possible correlation between biodosimetry and standard image-based dosimetry	(69)
Thyroid cancer (*n* = 24)	[^131^I]NaI	Before treatment and after 3–4 days	Elevated frequency of chromosomal aberrations observed in re-treated patients before [^131^I]-therapy allows estimation of a cumulative dose received from all previous treatments	(70)
Thyroid cancer (*n* = 8)	[^131^I]MIBG	Before treatment and after the last *in vivo* assay measurement (when iodine was near the detection limit—83–102 days post-administration)	Significant correlation with the administered activity values and with 24 h [^131^iodine] whole-body retention, but not with the activities measured at 24 h in the thyroid region	(71)
Thyroid cancer and neck relapse or lung metastases (*n* = 18)	[^131^I]NaI	Before treatment and 4 days after each cycle	Both chromosomal painting and conventional cytogenetics underestimate the cumulative dose after repeated iodine-131 treatments	(72)
Thyroid cancer (*n* = 50)	[^131^I]NaI	Chronic effects for up to 2 years	Permit retrospective biological dosimetry for up to 2 years, after therapeutic exposure to iodine-131	(73)
Thyroid cancer (*n* = 30)	[^131^I]NaI	Before treatment and 4 days after	Close agreement between the two methodologies	(74)
Thyroid cancer (*n* = 11)	[^131^I]NaI	Before treatment and daily for 7 days post-treatment, followed by a sample on days 14 and 28.	Physical vs. cytogenetic estimates of the whole-body radiation doses: good agreement in patients whose thyroid glands had previously been ablated by radioiodine. In patients who had varying degrees of thyroid function: considerable differences (cytogenetic value always higher).	(75)
24-colour karyotyping and M-FISH				
mCRPC	[^223^Ra]RaCl_2_	Before and every 4 weeks prior to the next cycle	Models presented provide an initial estimation of the cumulative absorbed dose received by the blood during incremental IMRT fractions and [^223^Ra]RaCl_2_ injections	(68)

(IMRT) intensity-modulated radiotherapy, (mCRPC) metastatic castration-resistant prostate cancer, (MIBG) meta-iodobenzylguanidine.

Results confirmed that the DCA allows cumulative dose estimations from all previous treatments and permits retrospective biological dosimetry for up to 2 years after therapeutic exposure to iodine-131 (70, 73). Correlations between DCA biodosimetry and image-based MIRD dose calculations have been shown (69, 71, 72, 74). However, M'Kacher et al. reported that biodosimetry estimated doses were 2–4 times higher than those obtained by the MIRD method (72). Similarly, Lloyd et al. also noted more chromosome aberrations compared to image-based estimates of the whole-body radiation doses in patients who had varying degrees of thyroid function. These differences in biological and image-based dosimetry might be due in part to the non-uniform irradiation of PBLs by local sources of activity in the thyroid and liver (75). Interestingly, another study showed a correlation between biodosimetry and the administered activity values as well as with iodine-131 total whole-body retention at 24 h post-administration (71).

Alternatively, Erselcan and co-workers applied the sister chromatid exchange (SCE) method to investigate acute and late chromosomal damage in the PBLs after [^131^I]NaI therapy. A significant difference in the number of SCEs was observed during the basal (before the start of treatment), acute (third day of therapy), and late (6 months) periods in patients treated with radioiodine (78). Another approach to quantifying the spectrum of chromosome-type aberrations is using 24-colour karyotyping and M-FISH. These assays provided initial estimates of the cumulative radiation dose received during a combination of intensity-modulated radiation therapy (IMRT) and radium-223 dichloride injections. Interestingly, blood samples exposed to this mixed radiation source were analysed to estimate the ratio of cells containing damage consistent with high-LET exposure and low-LET exposure. The assumption was made that all IMRT induced aberrations would be simple chromosomal exchanges and that those originating from radium-223 would be more complex. However, this method needs further investigation (68).

### 3.2. Biodosimetry using the cytokinesis block micronucleus assay

**Figure 4:**
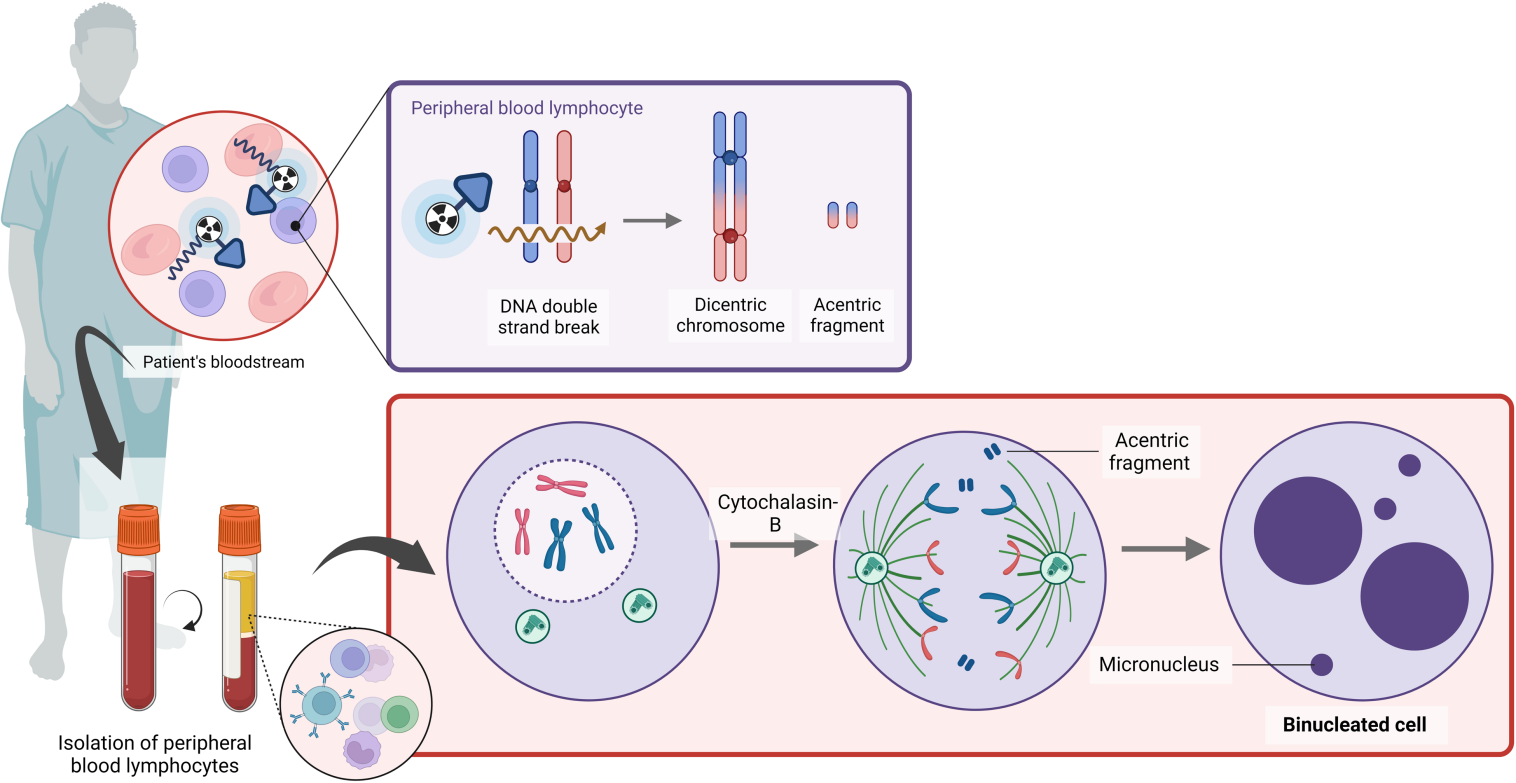
A schematic overview of the cytokinesis block micronucleus assay (created with BioRender.com).

The CBMN assay has been proven to be a useful tool for triage biodosimetry in large-scale nuclear incidents due to its simplicity of scoring, which requires less technical expertise (Figure 4). The whole-body dose assessment using the CBMN assay was evaluated in thyroid cancer patients upon iodine-131 therapy; however, a wide variation in micronucleus (MN) counts was noted (Table 3) (65, 79–87). Gutiérrez et al. noted that the number of MN increased in a dose-dependent manner in patients treated with iodine-131 (86). A reasonable correlation between the MIRD whole-body absorbed dose and the CBMN method was found by Monsieurs et al. (R = 0.87), while this correlation was limited in the study of Ozdal et al. at low radioiodine doses (88, 79). Hence, the applicability of the CBMN could be limited to high therapeutic doses (79).

**Table 3:** Overview of clinical studies performing biodosimetry in RLT patients using the CBMN.

Patient population	Radiopharmaceutical	Timing		Ref
Thyroid cancer (*n* = 47)	[^131^I]NaI	Pre- and 3 d after	The relationship between the MIRD and MN methods is limited. MN whole-body absorbed doses showed moderate correlation with administered activities in low doses and had significantly different and fluctuating values as compared to the MIRD method	(79)
Thyroid cancer (*n* = 25)	[^131^I]NaI	3 d after	Estimated blood dose after 3 d of exposure was 0.73 Gy (0.197 mGy/MBq). The Bayesian method for analysing chromosomal damage seems useful when the total count was close to or lower than the background level	(80)
Thyroid cancer (*n* = 5)	[^131^I]NaI	Pre- and 1 month after	The average frequency of MN before and after increased by more than double	(81)
Thyroid cancer (*n* = 22)	[^131^I]NaI	Pre- and 1 week after	Compared with the MN of all PBLs, the MN among specifically B cells may more sensitively detect cytologic radiation damage	(82)
Thyroid cancer (*n* = 11)	[^131^I]NaI	Pre- and 1,6,24 mo after	Investigated the induction and persistence of an adaptive response in PBLs	(83)
Neuroblastoma and carcinoid tumours (*n* = 22^a^)	[^131^I]MIBG	Pre- and 1 week after	ETBD using the MN assay was correlated with MIRD: important inter-individual variability in the total body dose, with the possibility of high dose values, suggests the necessity of individual dosimetry	(65)
Thyroid cancer (*n* = 20)	[^131^I]NaI	Pre- and 3–7 and 20–40 d after	MTT is more reliable than the MN test for evaluating induced lymphocyte damage	(84)
Thyrotoxicosis (*n* = 31) Thyroid cancer (*n* = 8)	[^131^I]NaI	Pre- and 1 week after	No relationship between administered doses and absorbed doses of irradiation on the basis of MN frequency in hyperthyroidism patients	(85)
Thyroid cancer (*n* = 39)	[^131^I]NaI	Pre- and 1 week, 6 months and 1 year after	A twofold increase in the frequency of MN was seen 1 week after therapy. Although this value decreased across time, the MN frequency obtained 1 year later remained higher than the value found before it	(86)
Thyroid cancer (*n* = 25)	[^131^I]NaI	1 week after	Relatively low frequency of MN induced and lack of significant effect on the frequency of MN with cumulative ^131^I supported the contention that short-term nonstochastic damage is minimal and reversible	(87)
Neuroblastoma (*n* = 18) Carcinoid tumour (*n* = 4)	[^131^I]MIBG	Pre- and 1 week after	MN assay only performed in 14/22 patients, PBL division inhibition caused by previous chemotherapy and dilution due to blood transfusion. MN increased significantly, greater than [^131^I]NaI or strontium-89 therapy, and lower than ERBT.	(88)

ETBD, equivalent total body dose; MIRD, medical internal radiation dosimetry; MN, Micronucleus; MIBG, meta-iodobenzylguanidine; MTT, tetrazolium salt 3-(4,5-dimethylthiazol-2-yl)-2,5-diphenyl tetrazolium bromide test, peripheral blood lymphocytes (PBLs).

^a^ Only evaluable in 14 due to cell division inhibition (due to chemo).

### 3.3. γ-H2AX/53BP1 foci assay

**Figure 5:**
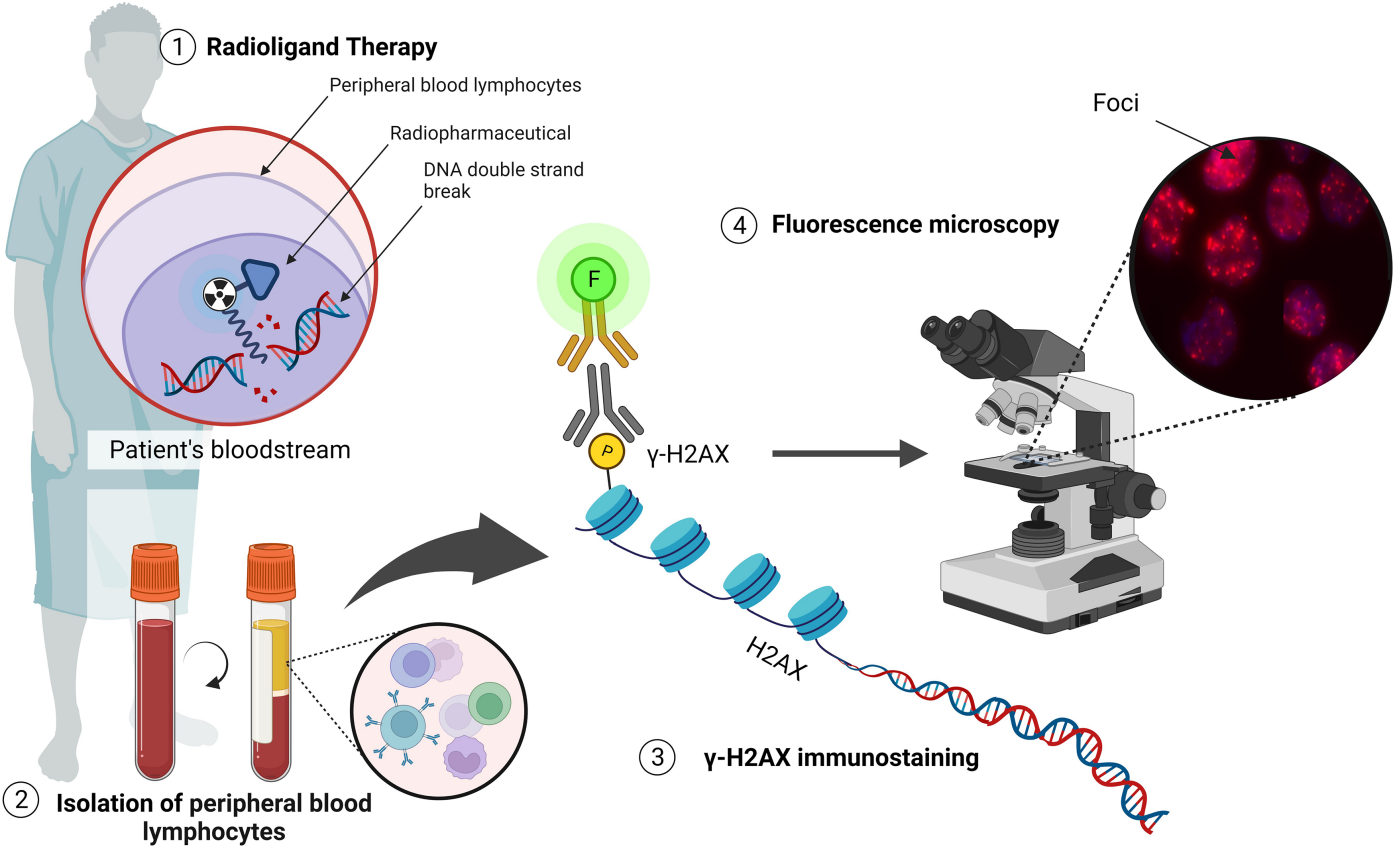
The detection of DNA DSBs via the γ-H2AX assay (illustrated with Biorender.com).

One of the earliest steps in the recognition of DNA DSB is the phosphorylation of the histone variant H2AX, which can be detected using fluorescently labelled antibodies (Figure 5). With small volumes of blood, a high sensitivity is achieved if applied within a few hours after exposure (as low as 10 mGy) (89, 90). The use of an automated microscopy platform to score the number of γ-H2AX foci per cell allows for a standardised analysis with a significant decrease in the turn-around time, as explained in our previous methodology paper (49). In addition to immunofluorescent staining of γ-H2AX, alternative DNA repair proteins can be used to evaluate co-localising fluorescent DNA repair proteins, which can improve the sensitivity of the assay, or to identify the involvement of different DNA DSB repair pathways. An alternative DNA repair protein that is commonly used in PBLs, is the p53 binding protein 1 (53BP1) (90–93). This is another important DNA DSB-responsive protein that promotes the repair of DNA DSB by nonhomologous end-joining while preventing homologous recombination (94, 95).

Studies have investigated radiation-induced DSBs during RLT by quantifying γ-H2AX foci or colocalizing γ-H2AX + 53BP1 foci (Table 4) (96). These include patients that received radioiodine therapy (97–99), ^177^Lu-labelled somatostatin-targeting peptide (DOTA-TATE/NOC) therapy (50, 96, 100), as well as [^177^Lu]Lu-PSMA (55). In general, an increase in the average number of radiation-induced foci was observed in the first hours after administration of the radiopharmaceutical, followed by a decrease due to DNA repair and a decreasing dose rate (55). Lassmann et al. concluded that γ-H2AX and 53BP1 foci are useful markers for detecting radiation exposure after RLT, even for absorbed doses to the blood below 20 mGy (97, 101). The potential of quantifying γ-H2AX in PBLs for predicting both individual subclinical haematotoxicity and tumour response to somatostatin receptor-targeted radioligand therapy was demonstrated by Derlin T et al. and Denoyer et al. where subclinical hematotoxicity was associated with γ-H2AX and 53BP1 foci formation (101, 102).

**Table 4:** Overview of clinical studies performing biodosimetry in RLT patients using the *γ*H2AX assay.

Patient population	Radio pharmaceutical	Timing post-administration	Main result	Ref
mCRPC (*n* = 20)	[^177^Lu]Lu-PSMA	Pre- and 1 h and 24 h after	Baseline 53BP1 foci demonstrated borderline significance for predicting progression-free survival	(104)
mCRPC (*n* = 9)	[^223^Ra]RaCl_2_	Pre- and 1.5, 3, 4, 24, 48 h (or 96 h) and 土 4 weeks after	May serve as a biomarker discriminating *α*- from *β*-emitters based on damage geometry	(100)
Advanced Gastroenteropancreatic NET (*n* = 21)	[^177^Lu]Lu-DOTA-TATE	Pre- and 1 h and 24 h after	May hold promise for predicting subclinical hematotoxicity and early treatment response	(102)
mCRPC (*n* = 17)	[^223^Ra]RaCl_2_	Pre- and before each therapy cycle (4 week interval)	The number of *γ*H2AX foci per cell was not changed in dependence on the therapy cycles	(103)
Prostate cancer (*n* = 16)	[^177^Lu]Lu-PSMA	Pre- and 1, 2, 3, 4, 24, 48 and 96 h after	Time- and dose-dependency of DSB induction and repair in peripheral blood leukocytes	(55)
Thyroid cancer (*n* = 20)	[^131^I]NaI	Pre- and 0.5, 1, 2, 3, 4, 24, 48 and up to 168 h after	A dose-response relationship is demonstrated, and an analytic function describes the time course of the *in vivo* damage response	(98)
NET (*n* = 11)	[^177^Lu]Lu-DOTA-TATE	Pre- and up to 72 h after	Kinetics of γ-H2AX foci in PBLs. γ-H2AX can be exploited as a biomarker of PBL cytotoxicity	(101)
NET (*n* = 16)	[^177^Lu]Lu-DOTA-TATE/TOC	Pre- and 1, 2, 3, 4, 24 and 48 h after	The average number of γ-H2AX foci and the absorbed dose into the blood may be used to obtain data on the individual dose-response relationships *in vivo*	(50)
Thyroid cancer (*n* = 15)	[^131^I]NaI	Pre- and 4 d after	May detect radiation-induced DNA damage associated with I-131 therapy, and may facilitate estimation of the radiation doses absorbed	(99)
Thyroid cancer (*n* = 26)	[^131^I]NaI	Pre- and 2, 24, 48, 72, 96 and 120 −144 h after	Useful markers for detecting radiation exposure after radionuclide incorporation, even for absorbed doses to the blood below 20 mGy	(97)

DSB, double strand break; mCRPC, metastatic castration-resistant prostate cancer; NET, neuroendocrine tumours; PBL, peripheral blood lymphocytes; PRRT, peptide receptor radionuclide therapy; PSMA, prostate-specific membrane antigen.

Interestingly, Schumann et al. used this method for the detection of α-particle-induced DNA damage upon [^223^Ra]RaCl_2_ therapy (blood samples up to 4 weeks post-therapy) and concluded that there is potential to discriminate α- from β-emitters based on damage geometry (100). However, Runge et al. could not detect *α*-particle tracks when 4 weeks lapsed after the administration of the therapy (103). In the recent prospective study of Widjaja et al.*,* low baseline γ-H2AX and 53BP1 markers in PBLs tended to predict poor outcomes in metastatic castration-resistant prostate cancer (mCRPC) patients undergoing [^177^Lu]Lu-PSMA RLT (104).

In addition to a biodosimetry tool, the γH2AX foci assay can investigate the time- and dose-dependency of DNA DSBs induction and repair in PBLs of cancer patients during RLT to obtain data on the individual dose–response relationships *in vivo* (50, 55). This biodosimetry approach could be used as an *in vivo* marker to assess individual radiosensitivity and normal-tissue toxicity after extended PRRT of NETs (101, 105). In addition to immunodetection, O'Neill et al. concluded that imaging via [^111^In]In-anti-γH2AX-TAT SPECT was able to monitor DNA damage in CA20948 somatostatin receptor–positive tumour xenografts after [^177^Lu]Lu-DOTA-TATE therapy. [^111^In]In-anti-γH2AX-TAT SPECT seems to be a suitable biodosimeter to predict the effectiveness of this RLT and could measure the dose-response within each tumour within each patient (56, 96). The presence of γH2AX foci has also been shown to correlate with somatostatin receptor 2 expression in neuroendocrine tumours (*ex vivo*), as a surrogate for the [^177^Lu]Lu-DOTA-TATE activity that would be accumulating at certain areas of the tumours to offset its radiation dose (106).

### 3.4. Biodosimetry using gene transcript analysis

**Figure 6:**
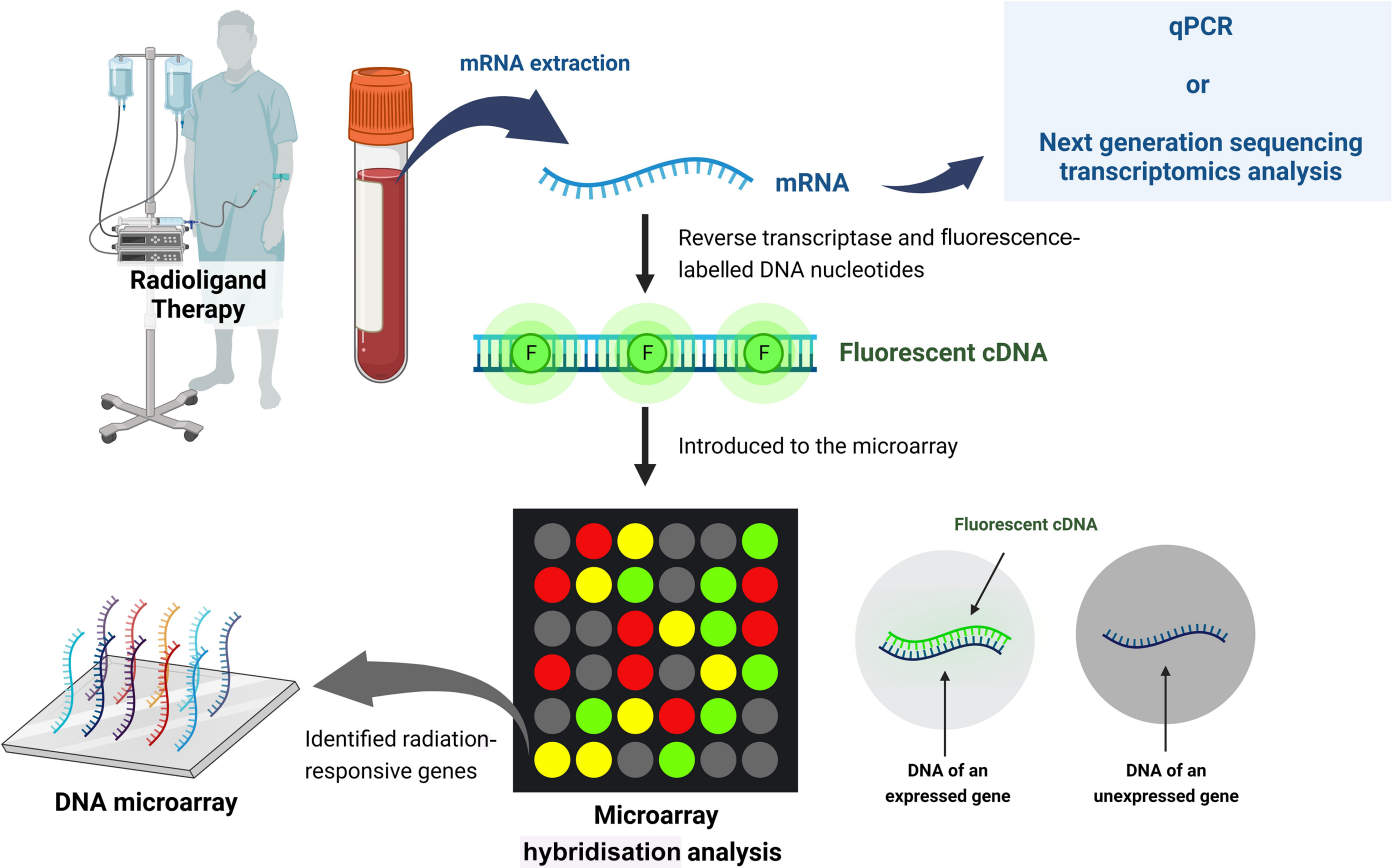
Schematic of biodosimetry using gene transcript analysis (created with BioRender.com).

Investigating the modulation of a select suite of genes enables the establishment of a diagnostic model for a patient’s or exposed individual’s absorbed dose in comparison to imaging-based dose calculations (65). This leads to the application of a biomarker based on gene transcript analysis, which can be used for dose estimations present in the whole blood of patients receiving RLT. Molecular protein or gene expression radiation exposure markers have been applied for retrospective biodosimetry and the prediction of acute health effects in external exposure accidents. This method is user-friendly due to its acute applicability (1–3 days after irradiation) and scalability for high-throughput applications (Figure 6) (107).

Microarray hybridization analysis provides an attractive avenue for the discovery of potentially informative radiation-exposure-sensitive gene expression profiles. Once validated, a set of biomarker genes could be developed into a simple and rapid assay on a sensitive quantitative real-time PCR multiplex platform (108). In 2017, Li et al. identified and validated 35 candidate radiation-responsive genes for human biodosimetry. These genes are involved in response to DNA damage, cell proliferation, cell cycle regulation, and DNA repair, with the top-enriched pathway being the well-studied p53 signalling pathway (109). Genes identified as promising biomarkers for biological dosimetry include for example, FDXR, DDB2, MDM2, ACTA2, ASCC3, BAX, AEN, BBC3, CDKN1A, CCNG1, GADD45, MDM2, and PCNA (110–113).

Edmonson et al. proposed a gene expression-based biodosimetry model using peripheral blood from neuroblastoma patients treated with ^131^I-labelled metaiodobenzylguanidine ([^131^I]MIBG) (Table 5). Three of the transcripts (CDKN1A, BAX and DDB2) explained over 98% of the variance in the modulation to gene expression over the 96 h post-therapy. Of major importance were the dose-dependent and time-dependent responses on gene expression, considering the exponentially decaying targeted radionuclide (65). There is currently an ongoing trial called GENEBIOLuNET, with the aim of exploring molecular biomarkers for [^177^Lu]Lu-DOTA-TATE NET therapy by studying gene transcript expression variations induced by this therapy (NCT03667092) (clinicaltrials.gov) (114).

**Table 5:** Overview of clinical studies exploring biodosimetry using gene transcript analysis.

Patient population	Radiopharmaceutical	Biodosimetry assay	Main result	Ref
Neuroblastoma (*n* = 40)	[^131^I]MIBG	GE transcript analysis -2.5 ml per time point: baseline, 72 h and 96h	Analysed 10 genes: CDKN1A; FDXR; GADD45A; BCLXL; STAT5B; BAX; BCL2; DDB2; XPC; and MDM2.Three of the transcripts (CDKN1A, BAX and DDB2) explained over 98% of the variance in the modulation of gene expression over the 96 h post-therapy	(65)
Neuroendocrine Tumours	[^177^Lu]Lu-DOTA-TATE	gene/miRNA transcripts before + 6 mo after + after 2 injections and add end of therapy	Recruiting	(114)

GE, Gene expression; MIBG, meta-iodobenzylguanidine.

Similar to the other biodosimetry assays, several challenges related to the use of transcriptomics in radiation biodosimetry have recently been published, including individual variations in gene expression and potential confounding factors (112). However, the main difficulty lies in the highly dynamic and transient nature of the signal, hence the time span between exposure and measurement is pivotal for the correct dose prediction (115).

## 4. Discussion. Can biodosimetry be integrated into the clinic?

**Figure 7:**
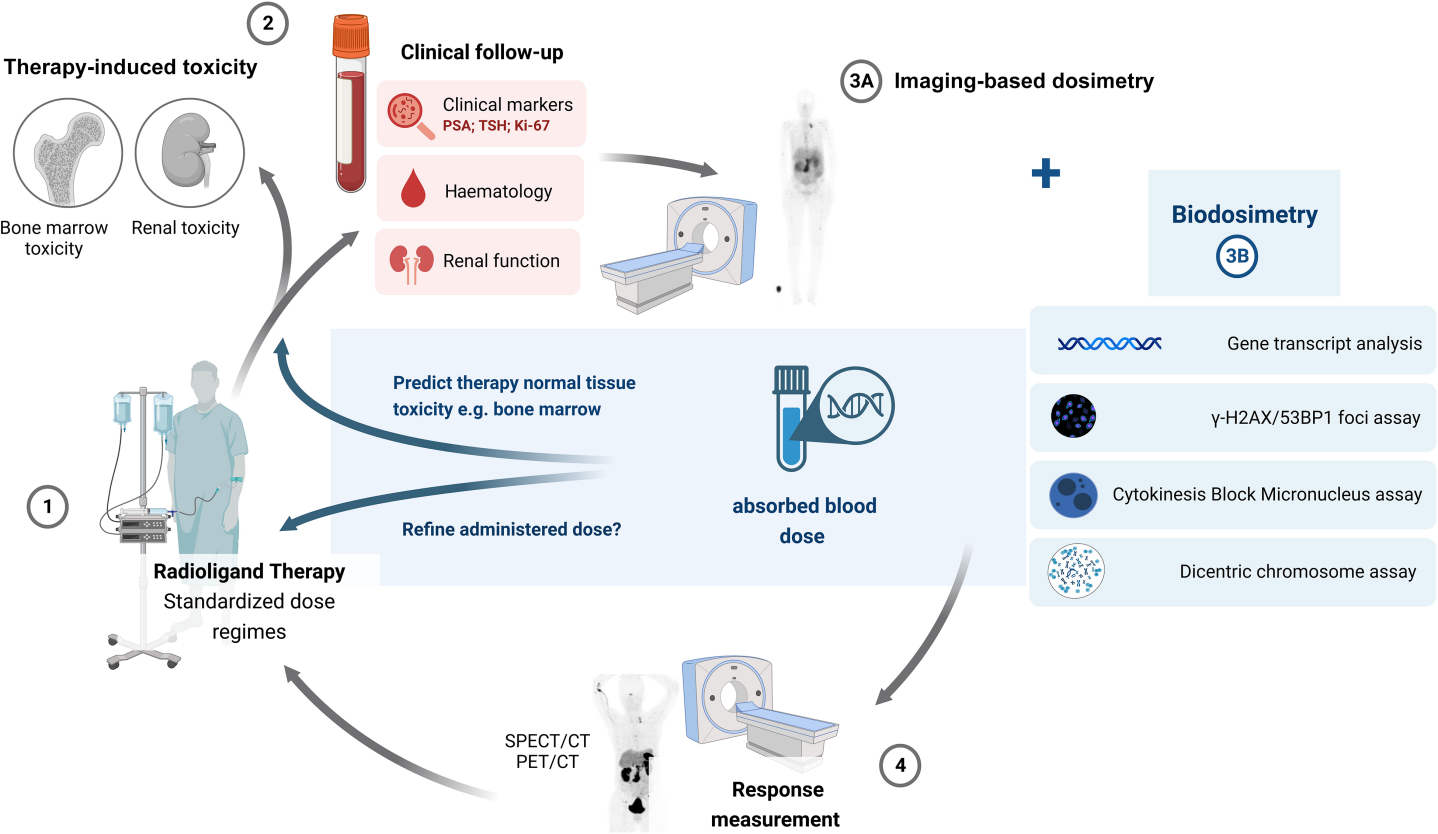
A proposed updated scheme incorporating biodosimetry-based investigations in radioligand therapy.

To realise the full potential of personalised theranostics, more advances are needed in the field of dosimetry for RLT. Image-based 3D dose MIRD calculations (planar or SPECT/CT) are not always available or incorporated to adjust the patient dose. In addition, bone marrow image-based dosimetry lacks accuracy, and its link to late-stage biological changes (such as kidney damage) is unknown (56, 116). In 2014, a meta-analysis showed a correlation between the delivered absorbed dose and the therapeutic response, indicating that dosimetry-based personalised treatments would improve prognosis and increase survival. However, the limited availability of published data on absorbed dose-effect relationships in RLT and the increasing role of radiobiological modelling in clinical data were also highlighted (117). As stated by Aerts et al., there is limited radiobiology data available from the nuclear medicine clinic. This is due to the inherent characteristics of RLT procedures (dose, dose rate and heterogeneous conditions treated) which make extrapolation from ERBT less useful. Incorporating biodosimetry-based investigations in clinical nuclear medicine trials, as shown in Figure 7, would increase RLT data availability. Although the integration of radiobiology assays into the clinic (e.g., microscopes or missing know-how), the associated cost, resources, and patient load (time per measurement) could hamper implementation, once the laboratory set-up is accomplished, the assays can be performed at a limited cost with results within a day to a few days (118).

Biodosimetry-based dose estimations will not provide a direct measurement of the tumour or organ-absorbed dose or whole-body dose but refer to an absorbed blood dose. It is still to be determined what the ideal dose parameter is (blood dose, marrow dose or total body dose) for which biodosimetry assays should be investigated and applied (61). The homogeneity of the irradiation of PBLs is very dependent on the radiopharmaceutical, since most are very organ-specific, limiting a dose correlation with the dose in the organ or target tissue. In addition, individual variations need to be considered, such as heterogeneity in biokinetics between patients (61). However, there is potential for biodosimetry to provide a measurement of the radiation dose received by the total blood volume and vascular walls, which can be used as a surrogate marker for the absorbed dose to the bone marrow. Biodosimetry could be used to overcome the difficulties of image-based bone marrow dosimetry due to its complex geometry and the presence of tissue inhomogeneities (101, 102, 119). However, more research is needed on their potential to predict hematotoxicity, on how these biomarkers correlate with image-based dose estimations, and their ability to accurately reflect the patient's administered dose.

Biodosimetry methods could also be particularly informative when image-based dosimetry or MIRD is not applicable (e.g., alpha-emitting radionuclides like actinium-225) (120). In the field of targeted alpha radionuclide therapy (TAT) for instance, real-time dosimetry is still problematic. Issues concerning image-based dosimetry for TAT include uncertainties on the relative biological effectiveness (RBE) value (which varies around 5), sub-organ localization of activity, relocation of daughters, e.g., alpha-emitting daughters of actinium-225, and a low count rate for imaging (typical therapeutic activity is 100 uCi to a few mCi). Here, biodosimetry could play a crucial role in TAT dosimetry to establish the biological dose after the first fraction (121). Advances are also needed in precision dosimetry at the cell or even sub-cellular level for alpha-particle emitters (118).

Many studies have shown that the range of the published estimates of blood doses after administration of 3.7 GBq iodine-131 (0.15–0.85 Gy), based on standard dose coefficients from ICRP and on individual dose studies, overlaps well with the range of 0.27–0.73 Gy estimated by means of cytogenetic assays, such as DC, MN or FISH translocations (61, 71–74, 80, 87, 97, 122–124). However, this review shows that the current data on the application of radiation biomarkers for biodosimetry in RLT and their correlation with image-based dosimetry remains limited. The majority of studies report a significant increase in DC and MN after RLT therapy; however, a correlation with an individual (image-based) estimation of the internal radiation dose is often missing and the research groups only compare the administered activity, which might be misleading. Compared with external exposures, biodosimetry of internal exposures, as in RLT, is more complex. Irradiation of the body is spatially inhomogeneous with preferential uptake in specific organs and tissues, prolonged over large periods and variable over time (61). RLT is characterised by prolonged irradiation with a permanently decreasing dose rate after administration of the radionuclide due to the biological and physical half-life of the radionuclide. Therefore, the dose rate will affect the frequency of chromosome-type aberrations due to DNA damage repair taking place during irradiation. The latter makes it cumbersome to use existing DCA dose-response curves to perform dose estimations, which are usually generated with reference radiation qualities, such as 250 kVp x-rays or Cobalt-60 gamma-rays at normal EBRT dose rates. It is plausible that the gamma emissions of radionuclides could be comparable to EBRT, but the dosimetry of shorter-ranging alpha and beta irradiation is different. Even if *in vitro* dose effect curves with different radionuclides could be performed, the simulation of radionuclide incorporation is a challenge because of this declining dose rate (61). The importance of dose-rate effects was confirmed using cytogenetic biodosimetry upon radioiodine therapy. Dose estimates were about 1.7-fold higher than those disregarding the effect of exposure duration. Hence, in re-treated patients, a neglected dose-rate effect can result in an underestimation of the cumulative whole-body dose by a factor ranging from 2.6 to 6.8 (70). Absorbed dose rate-dependency of *γ*-H2AX foci was also confirmed upon [^177^Lu]Lu-PSMA therapy and radioiodine therapy (55, 97). A decrease in the number of *γ*-H2AX foci was observed at later time points, despite the increasing absorbed dose into the blood (55).

Although the DCA has been the gold standard method in biodosimetry for decades, it has disadvantages for introduction in the theranostics clinic. The DCA method is time-consuming, laborious, and requires well-trained personnel for scoring. To accelerate the DCA, automated systems have been developed (125–127); several studies have demonstrated the possibility of scoring a limited number of cells (20–50) (128, 129) and a simplification by applying telomere and centromere fluorescence *in situ* hybridization has been proposed (115, 130). Despite the existence of automated microscopy platforms for the quantification of chromosome-type aberrations, improvements to automated systems with software-driven dose estimation are needed for high-throughput analysis. Another downside of the DCA is that the assay is dependent on the stimulation of PBLs, which is often challenging in RLT patients, and it takes on average about 52 h to get a result once stimulation is successful (63). The foci assay and gene expression assays are faster since they do not depend on the stimulation of PBLs and can provide results within the first 24 h. In addition, some uncertainties remain a topic of debate, such as the effects of individual differences in radiosensitivity on biological dose-response curves and the influence of radiation dose rate on “Dicentric chromosome + centric rings” in nuclear medicine applications. This led to revisions of the IAEA Manual 2011 in 2022 (131).

For the CBMN assay, it must be kept in mind that other genotoxic stresses besides radiation also induce DNA fragmentation, which could appear as MN in the cytoplasm of the cell. MN are also gender-specific and influenced by age (5, 132). This is particularly challenging in combined treatment strategies in RLT patients. However, compared to the DCA, the CBMN assay is less complex and requires less technical expertise. It should, however, be noted that performing cytogenetic assays, such as the DCA and CBMN assays, can be hampered due to cell division inhibition caused by previous chemotherapy treatments and lymphocyte dilution due to blood transfusions given shortly after RLT (88). Hence, stimulating the PBLs for both the DCA and CBMN assays might be challenging. Stimulation is not an issue for the γ-H2AX and gene expression assays. Compared to cytogenetic techniques, the γ-H2AX assay does not allow for precise retrospective biodosimetry and long-term effects evaluation since the repair half-life of DNA DSBs is relatively fast and the number of radiation-induced DNA DSBs will often return to background values after 24 h in PBLs. However, recent results confirm that the γ-H2AX assay could be exploited in somatostatin receptor-targeted radionuclide therapy as a biomarker of PBL cytotoxicity and early treatment response. The authors highlight that long-term follow-up studies investigating whether elevated residual γ-H2AX values are associated with acute myelotoxicity and secondary blood malignancy may be worthwhile (101, 102).

Both the sensitivity and the time dependency of the different biodosimetry assays should be considered. The cytogenetic assays are less radiation-sensitive compared to the γ-H2AX assay. The DCA assay has a threshold for whole-body dose of 0.1–0.2 Gy based on analysis of 1,000 metaphase spreads (133). For the γ-H2AX assay, guidelines for radiation emergencies (external exposure) have documented a minimum detectable dose increasing from a few mGy for a sample taken within 1 h after the exposure to approximately 0.5 Gy for a lag time of 2 days between exposure and sampling (134). Repair kinetics will vary significantly among individuals, and in the case of RLT, the protracted exposure leads to cumulative damage over days after the injection of the radiopharmaceutical. The change in gene expression post-irradiation is also time-dependent. No consensus with regards to the lag time has been reached to date, with literature reporting lag times from 4 h up to 3 days post-treatment. In the study of Edmonson et al., at 96 h after (^131^I)MIBG treatment, the modulation in gene expression was still significant enough to discriminate between exposed and unexposed samples using a selected gene transcript panel. This study also confirmed a time-dependent response of the gene expression biodosimetry model, most likely directly related to the exponentially decaying but persistent radiation field acting on the PBLs. This is in contrast with EBRT, where, as the time lapse increases after radiation exposure, the expression of differentially expressed genes decreases. Interestingly, prior RLT treatment cycles were not a confounding factor (65).

This review focused on the potential of biomarkers for biodosimetry, excluding the potential of the biomarkers as a predictive tool to determine a patient’s radiosensitivity prior to the first cycle of RLT or to assess the DNA repair capacity of each patient. If the radiosensitivity of each patient could be determined with a biomarker, then the necessary injected dose could be refined for every cycle of TRT to avoid therapy-induced toxicity (22, 96).

## 5. Conclusion

Despite the availability of biodosimetry during the last decades, biodosimetry has not found its way to the nuclear medicine clinic yet. Its potential lies in complementing MIRD to allow more personalised RLT dose estimations, in particular in case of non-theranostic radionuclides, and as a marker for bone marrow toxicity. Recent results confirm that biodosimetry could be exploited in RLT as a biomarker of PBLs cytotoxicity and early treatment response. Long-term, large cohort follow-up studies are, however, needed to confirm the correlation between radiation biomarkers in PBLs and acute myelotoxicity and secondary blood malignancy (101, 102). Before biodosimetry can be implemented in the nuclear medicine clinic, continuous developments of high-throughput biodosimetry techniques and clinical trials confirming its potential are needed. In addition, this requires a stronger interaction between radiochemists, radiopharmacists, radiobiologists, medical physicists, and physicians, as stated by the EANM (118). The most time efficient biodosimetry methods with regard to their introduction into the nuclear medicine clinic are most probably the *γ*-H2AX assay with automatic scoring or the analysis of changes in gene expression levels and the development of a biodosimetry gene signature at this stage (115). Dosimetry assays based on omics technologies, such as transcriptomics, proteomics, and metabolomics, hold great potential for large-scale dose estimations (101).

## References

[1] Bodei L Herrmann K Schoder H Scott AM Lews JS. Radiotheranostics in oncology: current challenges and emerging opportunities. Nat Rev Clinical Oncol. (2022) 19:534–50. 10.1038/s41571-022-00652-y 35725926 PMC10585450

[2] Sgouros G Bodei L McDevitt MR Nedrow JR. Radiopharmaceutical therapy in cancer: clinical advances and challenges. Nat Rev Drug Disc. (2020) 19:589–608. 10.1038/s41573-020-0073-9 PMC7390460 32728208

[3] Weber WA Czernin J Anderson CJ Badawi RD Barthel F Bengel F The future of nuclear medicine, molecular imaging and theranostics. J Nucl Med. (2020) 61(S2):263S–72S. 10.2967/jnmued.120.24532 33293447

[4] James S S Bednarz B Benedict S Buchsbaum JC Dewaraja Y Frey E Current status of radiopharmaceutical therapy. Int J Radiat Oncol Biol Phys. (2021) 109(4):891–901. 10.1016/j.ijrobp.2020.08.035 32805300 PMC8422258

[5] Sudprasert W Belyakov OV Tashiro S. Biological and internal dosimetry for radiation medicine: current status and future perspectives. J Radiat Res. (2022) 63(2):247–54. 10.1093/jrr/rrab119 34977921 PMC8944326

[6] Kratocwhil C Fendler WP Eiber M Baum R Bozkurt MF Czernin J EANM Procedures guidelines for radionuclide therapy with ^177^Lu-labelled PSMA-ligands (^177^Lu-PSMA-RLT). EJNMMI. (2019) 46(12):2536–44. 10.1007/s00259-019-04485-3 31440799

[7] Yadav MP Ballal S Sahoo RK Tripathi M Damle NA Shamim SA Long-term outcome of ^177^Lu-PSMA-617 radioligand therapy in heavily pre-treated metastatic castration-resistant prostate cancer patients. PLoS One. (2021) 16(5):30251375. 10.1371/journal.pone.0251375 PMC8109776 33970962

[8] Feuerecker B Tauber R Knorr K Heck M Behesthi A Seidl C Activity and adverse events of actinium-225-PSMA-617 in advanced metastatic castration resistant prostate cancer after failure of lutetium-177-PSMA. Eur Urology. (2021) 79(3):343–50. 10.1016/j.eururo.2020.11.013 33293081

[9] Swayamjeet S Amit S Ashwani S Pooja M Harinder G Singh J. Delayed nephrotoxicity after ^225^Ac-PSMA-617 radioligand therapy. Clin Nucl Med. (2022) 47(6):e466–7. 10.1097/RLU.0000000000004149 35353746

[10] Pelletier K Cote G Fallah-Rad N Johan R Kitchlu A. CKD After ^225^Ac-PSMA-617 therapy in patients with metastatic prostate cancer. Kidney Int Rep. (2021) 6(3):853–6. 10.1016/j.ekir.2020.12.006 33733002 PMC7938068

[11] Zaknun JJ Bodei L Mueller-Brand J Pavel ME Baum RP Horsch D The joint IAEA, EANM and SNMMI practical guidance on peptide receptor radionuclide therapy (PRRT) in neuroendocrine tumours. EJNMMI. (2013) 40(5):800–16. 10.1007/s00259-012-2330-6 PMC3622744 23389427

[12] Hope TA Abbott A Colucci K Bushnell DL Gardner L Graham WS NANETS/SNMMI procedure standard for somatostatin receptor-based peptide receptor radionuclide therapy with ^177^Lu-DOTATATE. J Nucl Med. (2019) 60(7):937–43. 10.2967/jnumed.118.230607 31263080 PMC12079155

[13] Poeppel TD Handkiewicz-Junak D Andreeff M Becherer A Bockisch A Fricke E EANM Guideline for radionuclide therapy with radium-223 of metastatic castration-resistant prostate cancer. EJNMMI. (2018) 45(5):824–45. 10.1007/s00259-017-3900-4 29234845

[14] Alexander C Bader JB Schaefer A Finke C Kirsch CM. Intermediate and long-term side effects of high-dose radioiodine therapy for thyroid carcinoma. J Nucl Med. (1998) 39(9):1551–4. 9744341

[15] Silberstein EB Alavi A Balon HR Clarke SEM Divgi C Gelfand MJ The SNMMI practice guideline for therapy of thyroid disease with ^131^I 3.0. J Nucl Med. (2012) 53(10):1633–51. 10.2967/jnumed.112.105148 22787108

[16] Luster M Clarke SE Dietlein M Lassmann M Lind P Oyen WJG Guidelines for radioiodine therapy of differentiated thyroid cancer. EJNMMI. (2008) 35(10):1941–59. 10.1007/s00259-008-0883-1 18670773

[17] Giammarile F Chiti A Lassmann M Brans B Flux G. EANM Procedure guidelines for ^131^I-meta-iodobenzylguanidine (^131^I-MIBG) therapy. EJNMMI. (2008) 35(5):1039–47. 10.1007/s00259-008-0715-3 18274745

[18] Wahl RL Sunderland J. Radiopharmaceutical dosimetry for cancer therapy: from theory to practice. J Nucl Med. (2021) 62(3):1S–2S. 10.2967/jnumed.121.263273 34857618

[19] Sundlöv A Sjörgreen-Gleisner K Svensson J Ljungberg M Olsson T Barnhardt P Individualized ^177^Lu-DOTATATE treatment of neuroendocrine tumours based on kidney dosimetry. EJNMMI. (2017) 44(9):1480–9. 10.1007/s00259-017-3678-4 PMC5506097 28331954

[20] Pouget JP Lozza C Deshayes E Boudousq V Navarro-Teulon I. Introduction to radiobiology of targeted radionuclide therapy. Front Medicine. (2015) 2(12):1–11. 10.3389/fmed.2015.00012 PMC4362338 25853132

[21] Satyamitra MM DiCarlo AL Hollingsworth BA Winters TA Taliaferro LP. Development of biomarkers for radiation biodosimetry and medical countermeasures research: current status, utility and regulatory pathways. Radiat Res. (2022) 197(5):514–32. 10.667/RADE-21-00157.1 34879151 PMC9119904

[22] O’Neill E Cornelissen B. Know thy tumour: biomarkers to improve treatment of molecular radionuclide therapy. Nucl Med Biol. (2022) 108–109 (44-53):108–9. 10.1016/j.nucmedbio.2022.02.004 35276447

[23] Ilan E Sandstorm M Wassberg C Sundin A Garske-Roman U Eriksson B Dose response of pancreatic neuroendocrine tumours treated with peptide receptor radionuclide therapy using 177Lu-DOTATATE. J Nucl Med. (2015) 56(2):177–82. 10.2967/jnumed.114.148437 25593115

[24] Lawhn-Heath C Hope TA Martinze J Fung EK Shin J Seo Y Dosimetry in radionuclide therapy: the clinical role of measuring radiation dose. Review Lancet Oncol. (2022) 23(2):E75–87. 10.1016/S1470-2045(21)00657-4 35114134

[25] Chen MK Yasrebi M Samii J Staib LH Doddamane I Cheng DW The utility of I-123 pretherapy scan in I-131 radioiodine therapy for thyroid cancer. Thyroid. (2012) 22(3):304–9. 10.1089/thy.2011.0203 22300251

[26] Yang J Codreanu I Servaes S Zhuang H. I-131 MIBG post-therapy scan is more sensitive than I-123 MIBG pretherapy scan in the evaluation of metastatic neuroblastoma. Nucl Med Commun. (2012) 33(11):1134–7. 10.1097/MNM.0b013e3283570ffe 22825037

[27] Bolch WE Eckerman KF Sgouros G Thomas SR Brill AB Fisher DR MIRD Pamphlet No. 21: a generalized schema for radiopharmaceutical dosimetry-standardization of nomenclature. J Nucl Med. (2009) 50(3):477–84. 10.2967/jnumed.108.056036 19258258

[28] O’Donoghue J Zanzonico P Humm J Kesner A. Dosimetry in radiopharmaceutical therapy. J Nucl Med. (2022) 63(10):1467–74. 10.2967/jnumed.121.262305 36192334 PMC12079709

[29] Huizing DMV de Wit-van der Veen BJ Verheij M Stokkel MPM. Dosimetry methods and clinical applications in peptide receptor radionuclide therapy for neuroendocrine tumours: a literature review. EJNMMI Res. (2018) 8(1):89. 10.1186/s13550-018-0443-z 30159614 PMC6115319

[30] Ljungberg M Celler A Konijnenberg MW Eckerman KF Dewaraja YK Sjögreen-Gleisner K MIRD Pamphlet No. 26: joint EANM/MIRD guidelines for quantitative ^177^Lu SPECT applied for dosimetry of radiopharmaceutical therapy. J Nucl Med. (2016) 57(1):151–62. 10.2967/jnumed.115.159012 26471692

[31] Stokkel MPM Junak DH Lassmann M Dietlein M Luster M. EANM Procedure guidelines for therapy of benign thyroid disease. EJNMMI. (2010) 37(11):2218–28. 10.1007/s00259-010-1536-8 20625722

[32] Erdogan M Sengul SS Cetin B Avici M Yagici S Ozkoç I The role of Ga^68^ PSMA PET/CT imaging in Lu^177^ PSMA treatment planning in metastatic castration- resistant prostate cancer. Ann Nucl Med. (2022) 36(6):562–9. 10.1007/s12149-022-01739-3 35397091

[33] Peters SMB Hofferber R Prive BM De Bakker M Gotthardt M Janssen M (^68^ga)Ga-PSMA-11 PET imaging as a predictor for absorbed doses in organs at risk and small lesions in (^177^Lu)Lu-PSMA-617 treatment. EJNMMI. (2022) 49(4):1101–12. 10.1007/s00259-021-05538-2 PMC8921092 34623453

[34] Kletting P Müller B Erentok B Schmaljohann J Behrendt FF Reske SN Differences in predicted and actually absorbed doses in peptide receptor radionuclide therapy. Med Phys. (2012) 39(9):5708–17. 10.1118/1.4747266 22957636

[35] Geenen L Nonnekens J Konijnenberg M Baatout S De Jong M Aerts A. Overcoming nephrotoxicity in peptide receptor radionuclide therapy using (^177^Lu)Lu-DOTA-TATE for the treatment of neuroendocrine tumours. Nucl Med Biol. (2021) 102-103:1–11. 10.1016/j.nucmedbio.2021.06.006 34242948

[36] Mittra ES. Neuroendocrine tumour therapy: ^177^Lu-DOTATATE. Nucl Med Mol Imaging. (2018) 211(2):237–467. 10.2214/AJR.18.19953 29949416

[37] Madsen MT Menda Y O’Dorisio TM O’Dorisio MS. Technical note: single time point dose estimation for exponential clearance. Med Phys. (2018) 45(5):2318–24. 10.1002/mp.12886 29577338 PMC5948162

[38] Hänscheid H Lapa C Buck AK Lassmann M Werner RA. Dose mapping after endoradiotherapy with ^177^Lu-DOTATATE/DOTATOC by a single measurement after 4 days. J Nucl Med. (2018) 59(1):75–81. 10.2967/jnumed.117.193706 28588150

[39] Siebinga H Privé BM Peters SMB Nagarajah J Dorlo TPC Huitema ADR Population pharmacokinetic dosimetry model using imaging data to assess variability in pharmacokinetics of ^177^Lu-PSMA-617 in prostate cancer patients. CPT Pharmacometrics Syst Pharmacol. (2023) 00:1–12. 10.1002/psp4.12914 PMC10431047 36760133

[40] Tronchin S Forster JC Hickson K Bezak E. Dosimetry in targeted alpha therapy: a systematic review: current findings and what is needed. Phys Med Biol. (2022) 67(9):09TR01. 10.1088/1361-6560/ac5fe0 35316802

[41] Essers M Mijnheer BJ. *In vivo* dosimetry during external photon beam radiotherapy. Int J Radiation Oncology Biol Phys. (1999) 43(2):245–59. 10.1016/S0360-3016(98)00341-1 10030247

[42] Costa AM Barbi GL Bertucci EC Ferreira H Sansavino SZ Colenci B *In vivo* dosimetry with thermoluminescent dosimeters in external photon beam radiotherapy. Appl Radiat Isot. (2010) 68(4–5):760–2. 10.1016/j.apradiso.2009.09.039 19819151

[43] Jaberi R Babaloui S Siavashpour Z Moshtaghi M Shirazi A Joya M 3D In vivo dosimetry of HDR gynecological brachytherapy using micro silica bead TLDs. J Appl Clin Med Phys. (2022) 23(9):e13729. 10.1002/acm2.13729 35946855 PMC9512342

[44] Yorke ED Williams LE Demidecki AJ Heidorn DB Roberson PL Wessels BW. Multicellular dosimetry for beta-emitting radionuclides: autoradiography, thermoluminescent dosimetry and three-dimensional dose calculations. Med Phys. (1993) 20(2):543–50. 10.1118/1.597050 8492763

[45] Gustafsson J Taprogge J. Theoretical aspects on the use of single-time-point dosimetry for radionuclide therapy. Phys Med Biol. (2022) 67(2):025003. 10.1088/1361-6560/ac46e0 34965519

[46] Hou X Brosch J Uribe C Desy A Böning G Beauregard JM Feasibility of single-time-point dosimetry for radiopharmaceutical therapies. JNM. (2021) 62(7):1006–11. 10.2967/jnumed.120.254656 33127625 PMC8882881

[47] Hardiansyah D Riana A Beer AJ Glatting G. Single-time-point dosimetry using model selection and nonlinear mixed-effects modelling: a proof of concept. EJNMMI Phys. (2023) 10(1):12. 10.1186/s40658-023-00530-1 36759362 PMC9911583

[48] Brosch-Lenz J Delker A Völter F Unterrainer LM Kaiser L Bartenstein P Towards single time point image based dosimetry of 177Lu-PSMA-617 therapy. JNM. (2023) 64(6):767–74. 10.2967/jnumed.122.264594 36657980 PMC10152120

[49] Nair S Carincross S Miles X Engelbrecth M Du Plessis P Bolcaen J An automated microscopic scoring method for the γH2AX foci assay in human peripheral blood lymphocytes. J Vis Exp. (2021) 178:e62623. 10.3791/62623 35001906

[50] Eberlein U Nowak C Bluemel C Buck AK Werner RA Scherthan H DNA Damage in blood lymphocytes in patients after ^177^Lu peptide receptor radionuclide therapy. EJNMMI. (2015) 42(11):1739–49. 10.1007/s00259-015-3083-9 PMC4554740 26048612

[51] Carrano AV Heddle JA. The fate of chromosome aberrations. J Theor Biol. (1973) 38(2):289–304. 10.1016/0022-5193(73)90176-8 4689998

[52] Lassmann M Eberlein U. Radiation dosimetry aspects of ^177^Lu. Curr Radiopharm. (2015) 8(2):139–44. 10.2174/1874471008666150313104212 25771372

[53] Hindorf C Glatting G Chiesa C Lindén O Flux G. EANM Dosimetry committee guidelines for bone marrow and whole-body dosimetry. EJNMMI. (2010) 37(6):1238–50. 10.1007/s00259-010-1422-4 20411259

[54] Hagmarker L Svensson J Rydén T van Essen M Sundlöv A Gleisner KS Bone marrow absorbed doses and correlations with hematologic response during ^177^Lu-DOTATATE treatments are influenced by image-based dosimetry method and presence of skeletal metastases. J Nucl Med. (2019) 60(10):1406–13. 10.2967/jnumed.118.225235 30902877 PMC6785794

[55] Schumann S Scherthan H Lapa C Serfling S Muhtadi R Lassmann M DNA Damage in blood leukocytes of prostate cancer patients during therapy with ^177^Lu-PSMA. EJNMMI. (2019) 46(8):723–1732. 10.1007/s00259-019-04317-4 PMC6584247 31028426

[56] O’Neill E Mosley M Cornelissen B. Imaging DNA damage response by γH2AX in vivo predicts treatment response to lutetium-177 radioligand therapy and suggests senescence as a therapeutically desirable outcome. Theranostics. (2023) 13(4):1302–10. 10.7150/thno.82101 36923536 PMC10008745

[57] Walrand S Barone R Pauwels S Jamar F. Experimental facts supporting a red marrow uptake due to radiometal transchelation in ^90^Y-DOTATOC therapy and relationship to the decrease of platelet counts. EJNMMI. (2011) 38(7):1270–80. 10.1007/s00259-011-1744-x 21318451

[58] Sapienza MT Willegaignon J. Radionuclide therapy: current status and prospects for internal dosimetry in individualized therapeutic planning. Clinics. (2019) 74(1):e835. 10.6061/clinics/2019/e835 31365617 PMC6644503

[59] Pellegrini G Siwowska K Haller S Antoine DJ Schibli R Kipar A A short-term biological indicator for long-term kidney damage after radionuclide therapy in mice. Pharmaceuticals. (2017) 10(2):57. 10.3390/ph10020057 28635637 PMC5490414

[60] Schüler E Larsson M Parris TZ Johansson ME Helou K Forssell-Aronsson E. Potential biomarkers for radiation-induced renal toxicity following ^177^Lu-octreotate administration in mice. Plos One. (2015) 10(8):e0136204. 10.1371/journal.pone.0136204 26287527 PMC4546116

[61] Giussani A Lopez MA Romm H Testa A Ainsbury EA Degteva M Eurados review of retrospective dosimetry techniques for internal exposures to ionising radiation and their applications. Radiat Environ Biophys. (2020) 59(3):357–87. 10.1007/s00411-020-00845-y 32372284 PMC7369133

[62] Ludovici GM Cascone MG Huber T Chierici A Gaudio P De Souza O Cytogenetic bio-dosimetry techniques in the detection of dicentric chromosomes induced by ionizing radiation: a review. Eur Physical J Plus. (2021) 136(5):482. 10.1140/epjp/s13360-021-01447-3

[63] International Atomic Energy Agency. Cytogenetic dosimetry: Applications in preparedness for and response to radiation, emergency preparedness and response. Vienna, Austria: IAEA (2011). Available at: https://www.iaea.org/publications/8735/cytogenetic-dosimetry-applications-in-preparedness-for-and-response-to-radiation-emergencies

[64] Raavi V Perumal V Paul SFD. Potential application of y-H2AX as a biodosimetry tool for radiation triage. Mutat Res Rev Mutat Res. (2021) 787:108350. 10.1016/j.mrrev.2020.08350 34083048

[65] Edmondson DA Kraski EE Kohlgruber A Koneru H Matthay KK Allen S Transcript analysis for internal biodosimetry using peripheral blood from neuroblastoma patients treated with (131)I-mIBG, a targeted radionuclide. Radiation Res. (2016) 186(3):234–44. 10.1667/RR14263.1 PMC5047008 27556353

[66] Wong K Siu LLP Ainsbury E Moquet J. Cytogenetic biodosimetry: what it is and how we do it. Hong Kong Med J. (2013) 19(2):168–73. Available at: https://www.hkmj.org/abstracts/v19n2/168.htm 23535678

[67] International Organization for Standardization. Radiation protection — performance criteria for laboratories performing cytogenetic triage for assessment of mass casualties in radiological or nuclear emergencies — general principles and application to dicentric assay. Geneva, Switzerland: ISO (2022). Available at: https://www.iso.org/standard/78139.html

[68] Bastiani I McMahon SJ Turner P Redmond KM McGarry CK Cole A Dose estimation after a mixed field exposure: radium-223 and intensity modulated radiotherapy. Nucl Med Biol. (2022) 106-107:10–20. 10.1016/j.nucmedbio.2021.12.002 34968973

[69] Chimeno JM Sebastià N Torres-Espallardo I Balaguer J Candela-Juan C Loaiza JL Assessment of the dicentric chromosome assay as a biodosimetry tool for more personalised medicine in a case of high risk neuroblastoma ^131^I-MIBG treatment. Int J Radiat Biol. (2019) 95(3):314–20. 10.1080/09553002.2019.1549755 30496023

[70] Khvostunov IK Saenko VA Krylov V Rodichev A Yamashita S. Cytogenetic biodosimetry and dose-rate effect after radioiodine therapy for thyroid cancer. Radiat Environ Biophys. (2017) 56(3):213–26. 10.1007/s00411-017-0696-3 28526978

[71] Nascimento ACH Lipsztein JL Corbo R Rebelo AMO. ^131^I Biokinetics and cytogenetic dose estimates in ablation treatment of thyroid carcinoma. Health Phys. (2010) 99(4):457–63. 10.1097/HP.0b013e3181c8f9ea 20838086

[72] M’Kacher R Schlumberger M Légal JD Violot D Béron-Gaillard N Gaussen A Biologic dosimetry in thyroid cancer patients after repeated treatments with iodine-131. J Nucl Med. (1998) 38(5):828–9. 9591584

[73] M’Kacher R Légal JD Schlumberger M Aubert B Beron-Gaillard N Gaussen A Sequential biological dosimetry after a single treatment with iodine-131 for differentiated thyroid carcinoma. J Nucl Med. (1997) 38(3):377–80. 9074522

[74] M’Kacher R Légal JD Schlumberger M Voisin P Auberg B Gaillard N Biological dosimetry in patients treated with iodine-131 for differentiated thyroid carcinoma. J Nucl Med. (1996) 37(11):1860–4. 8917193

[75] Llloyd DC Purrott RJ Dolphin GW Horton PW Halnan KE Scott JS A comparison of physical and cytogenetic estimates of radiation dose in patients treated with iodine-131 for thyroid carcinoma. Int J Radiat Biol Relat Stud Phys Chem Med. (1976) 30(5):473–85. 10.1080/09553007614551291 1087288

[76] Kavakli K Cogulu O Aydogdu S Ozkilic H Durmaz B Kirbiyik O Prospective evaluation of chromosomal breakages in hemophiliac children after radioisotope synovectomy with Yttrium^90^ and Rhenium^186^. Blood. (2008) 112(11):1219. 10.1182/blood.V112.11.1219.1219

[77] O'Duffy EK Oliver FJ Chatters SJ Walker H Lloyd D Edwards JCW Chromosomal analysis of peripheral lymphocytes of patients before and after radiation synovectomy with samarium-153 particulate hydroxyapatite. Rheumatology. (1999) 38(4):316–20. 10.1093/rheumatology/38.4.316 10378707

[78] Erselcan T Sungu S Ozdemir S Turgut B Dogan D Ozdemir O. Iodine-131 treatment and chromosomal damage: in vivo dose-effect relationship. EJNMMI. (2004) 31(5):676–84. 10.1007/s00259-003-1427-3 14747958

[79] Ozdal A Erselcan T Özdemir Ö Özguven Y Silov G Erdogan Z. Evaluation of the physical and biological dosimetry methods in iodine-131 treated patients. World J Nucl Med. (2018) 17(4):253–60. 10.4103/wjnm.WJNM_78_17 30505223 PMC6216729

[80] Serna A Alcaraz M Navarro JL Acevedo C Vicente V Canteras M. Biological dosimetry and Bayesian analysis of chromosomal damage in thyroid cancer patients. Radiat Prot Dosimetry. (2008) 129(4):372–80. 10.1093/rpd/ncm444 17951242

[81] Popova L Hadjidekova V Hadjieva T Agova S Vasilev I. Cytokinesis-block micronucleus test in patients undergoing radioiodine therapy for differentiated thyroid carcinoma. Hell J Nucl Med. (2005) 8(1):54–7. 15886755

[82] Watanabe N Kanegane H Kinuya S Shuke N Yokoyama K Kato H The radiotoxicity of ^131^I therapy of thyroid cancer: assessment by micronucleus assay of B lymphocytes. J Nucl Med. (2004) 45(4):608–11. 15073256

[83] Gill O M Oliveira NG Rodrigues AS Laires A Ferreira TC Limbert E Cytogenetic alterations and oxidative stress in thyroid cancer patients after iodine-131 therapy. Mutagenesis. (2000) 15(1):69–75. 10.1093/mutage/15.1.69 10640533

[84] Catena C Conti D Trenta G Righi E Breuer F Melacrinis FF Micronucleus yield and colorimetric test as indicators of damage in patients’ lymphocytes after ^131^I therapy. J Nucl Med. (2000) 41(9):1522–4. Available at: https://pubmed.ncbi.nlm.nih.gov/10994733/10994733

[85] Monsieurs MA Thierens HM van de Wiele CV Vral AM Meirlaen IA de Winter HA Estimation of risk based on biological dosimetry for patients treated with radioiodine. Nucl Med Commun. (1999) 20(10):911–7. 10.1097/00006231-199910000-00008 10528296

[86] Gutiérrez S Carbonell E Galofré P Creus A Marcos R. Cytogenetic damage after 131-iodine treatment for hyperthyroidism and thyroid cancer. Eur J Nucl Med. (1999) 26(12):1589–96. 10.1007/s002590050499 10638411

[87] Watanabe N Yokoyama K Kinuya S Shuke N Shimizu M Futatsuya R Radiotoxicity after iodine-131 therapy for thyroid cancer using the micronucleus assay. J Nucl Med. (1998) 39(3):436–40. Available at: https://pubmed.ncbi.nlm.nih.gov/9529288/9529288

[88] Monsieurs MA Thierens HM Vral A Brans B De Ridder L Dierckx RA. Patient dosimetry after 131I-MIBG therapy for neuroblastoma and carcinoid tumours. Nucl Med Commun. (2001) 22(4):367–674. 10.1097/00006231-200104000-00004 11338046

[89] Moquet J Barnard S Rothkamm K. Gamma-H2AX biodosimetry for use in large scale radiation incidents: comparison of a rapid ‘96 well lyse/fix’ protocol with a routine method. Peer J. (2014) 2:e285. 10.7717/peerj.282 24688860 PMC3961158

[90] Vandevoorde C Gomolka M Roessler U Samaga D Lindholm C Fernet M EPI-CT: in vitro assessment of the applicability of the γ-H2AX-foci assay as cellular biomarker for exposure in a multicentre study of children in diagnostic radiology. Int J Radiat Biol. (2015) 91(8):653–63. 10.3109/09553002.2015.1047987 25968559

[91] Chaurasia RK Shirsath KB Desai UN Bhat NN Sapra BK. Establishment of in vitro calibration curve for ^60^Co-γ-rays induced phospho-53BP1 foci, rapid biodosimetry and initial triage, and comparative evaluations with γH2AX and cytogenetic assays. Front Public Health. (2022) 10:845200. 10.3389/fpubh.2022.845200 36003625 PMC9393360

[92] Bucher M Duchrow L Endesfelder D Roessler U Gomolka M. Comparison of inexperienced operators and experts in *γ*H2A.X and 53BP1 foci assay for high-throughput biodosimetry approaches in a mass casualty incident. Int J Radiat Biol. (2020) 96(10):1263–73. 10.1080/09553002.2020.1793024 32673132

[93] Popp HD Brendel S Hofmann WK Fabarius A. Immunofluorescence microscopy of *γ*H2AX and 53BP1 for analyzing the formation and repair of DNA double-strand breaks. J Vis Exp. (2017) 129:56617. 10.3791/56617 PMC5755291 29155797

[94] Panier S Boulton SJ. Double-strand break repair: 53BP1 comes into focus. Nat Rev Mol Cell Biol. (2014) 15(1):7–18. 10.1038/nrm3719 24326623

[95] Escribano-Díaz C Orthwein A Fradet-Turcotte A Xing M Young JTF Tkáč J A cell cycle-dependent regulatory circuit composed of 53BP1-RIF1 and BRCA1-CtIP controls DNA repair pathway choice. Mol Cell. (2013) 49(5):872–83. 10.1016/j.molcel.2013.01.001 23333306

[96] O’Neill E Kersemans V Allen PD Terry SYA Torres JB Mosley M Imaging DNA damage repair in vivo after ^177^Lu-DOTATATE therapy. J Nucl Med. (2020) 61(5):743–50. 10.2967/jnumed.119.232934 31757844 PMC7198382

[97] Lassmann M Hänscheid H Gassen D Biko J Meineke V Reiners C In vivo formation of γ-H2AX and 53BP1 DNA repair foci in blood cells after radioiodine therapy of differentiated thyroid cancer. J Nucl Med. (2010) 51(8):1318–25. 10.2967/jnumed.109.071357 20660387

[98] Eberlein U Scherthan H Bluemel C Peper M Lapa C Buck AK DNA Damage in peripheral blood lymphocytes of thyroid cancer patients after radioiodine therapy. J Nucl Med. (2016) 57(2):173–9. 10.2967/jnumed.115.164814 26564321

[99] Doai M Watanabe N Takahashi T Taniguchi M Tonami H Iwabuchi K Sensitive immunodetection of radiotoxicity after iodine-131 therapy for thyroid cancer using γ-H2AX foci of DNA damage in lymphocytes. Ann Nucl Med. (2013) 27(3):233–8. 10.1007/s12149-012-0678-0 23264066

[100] Schumann S Eberlein U Lapa C Muller J Serfling S Lassmann M ɑ-particle-induced DNA damage tracks in peripheral blood mononuclear cells of (^223^Ra)RaCl_2_-treated prostate cancer patients. EJNMMI. (2021) 48(9):2761–70. 10.1007/s00259-020-05170-6 PMC8263441 33537837

[101] Denoyer D Lobachevsky P Jackson P Thompson M Martin OA Hicks RJ. Analysis of ^177^Lu-DOTA-octreotate therapy-induced DNA damage in peripheral blood lymphocytes of patients with neuroendocrine tumors. J Nucl Med. (2015) 56(4):505–11. 10.2967/jnumed.114.145581 25722453

[102] Derlin T Bogdanova N Ohlendorf F Ramachandran D Werner RA Ross TL Assessment of γ-H2AX and 53BP1 foci in peripheral blood lymphocytes to predict subclinical hematotoxicity and response in somatostatin receptor-targeted radionuclide therapy for advanced gastroenteropancreatic neuroendocrine tumors. Cancers. (2021) 13(7):1516. 10.3390/cancers13071516 33806081 PMC8036952

[103] Runge R Oehme L Grosche-Schlee S Braune A Freudenberg R Kotzerke J. Induction and rejoining of DNA double-strand breaks in the lymphocytes of prostate cancer patients after radium-223 treatment as assessed by the γh2AX foci assay. NuklearMedizin. (2019) 58(5):387–94. 10.1055/a-0974-3767 31387125

[104] Widjaja L Werner RA Krischke E Christiansen H Bengel FM Bogdanova N Individual radiosensitivity reflected by γ-H2AX and 53BP1 foci predicts outcome in PSMA-targeted radioligand therapy. EJNMMI. (2022) 50(2):602–12. 10.1007/s00259-022-05974-8 PMC9816192 36136101

[105] Ivashkevich A Redon CE Nakamura AJ Martin RF Martin OA. Use of the γ-H2AX assay to monitor DNA damage and repair in translational cancer research. Cancer Lett. (2012) 327(1-2):123–33. 10.1016/j.canlet.2011.12.025 22198208 PMC3329565

[106] Tamborino G Nonnekens J De Saint-Hubert M Struelens L Feijtel D de Jong M Dosimetric evaluation of the effect of receptor heterogeneity on the therapeutic efficacy of peptide receptor radionuclide therapy: correlation with DNA damage induction and in vivo survival. J Nucl Med. (2022) 63(1):100–7. 10.2967/jnumed.121.262122 33837068 PMC8717202

[107] Abend M Blakely WF Ostheim P Schuele S Port M. Early molecular markers for retrospective biodosimetry and prediction of acute health effects. J Radiol Prot. (2022) 42(1):010503. 10.1088/1361-6498/ac2434 34492641

[108] Amundson SA Grace MB Mcleland CB Epperly MW Yeager A Zhan Q Human *In vivo* radiation-induced biomarkers: gene expression changes in radiotherapy patients. Cancer Res. (2004) 64(18):6368–71. 10.1158/0008-5472.CAN-04-1883 15374940

[109] Li S Lu X Feng JB Tian M Liu QJ. Identification and validation of candidate radiation-responsive genes for human biodosimetry. Biomed Environ Sci. (2017) 30(11):834–40. 10.3967/bes2017.112 29216961

[110] Hladik D Bucher M Endesfelder D Oestreicher U. The potential of omics in biological dosimetry. Radiation. (2022) 2(1):78–90. 10.3390/radiation2010006

[111] Ghandhi SA Shuryak I Morton SR Amundson SA Brenner DJ. New approaches for quantitative reconstruction of radiation dose in human blood cells. Sci Rep. (2019) 9(1):18441. 10.1038/s41598-019-54967-5 31804590 PMC6895166

[112] Amundson SA. Transcriptomics for radiation biodosimetry: progress and challenges. Int J Radiat Biol. (2021) 99(6):1–9. 10.1080/09553002.2021.1928784 PMC10026363 33970766

[113] Ghandhi SA Smilenov LB Elliston CD Chowdhury M Amundson SA. Radiation dose-rate effects on gene expression for human biodosimetry functional and structural genomics. BMC Med Genomics. (2015) 8(1):22. 10.1186/s12920-015-0097-x 25963628 PMC4472181

[114] National Library of Medicine (US). Exploration of Molecular Biomarkers for Lu-177 DOTATATE Therapy in Midgut Neuroendocrine Tumor (GENEBIOLuNET). Identifier NCT03667092 (2018). Available at: https://clinicaltrials.gov/ct2/show/NCT03667092 (Accessed April 06, 2023).

[115] Macaeva E Mysara M de Vos WH Baatout S Quintens R. Gene expression-based biodosimetry for radiological incidents: assessment of dose and time after radiation exposure. Int J Radiat Biol. (2019) 95(1):64–75. 10.1080/09553002.2018.1511926 30247087

[116] Alsadi R Djekidel M Bouhali O O’Doherty J. Towards routine clinical use of dosimetry in (177Lu)-lu-PSMA prostate cancer radionuclide therapy: current efforts and future perspectives. Front Phys. (2022) 10:940677. 10.3389/fphy.2022.940677

[117] Strigari L Konijnenberg M Chiesa C Bardies M Du Y Gleisner KS The evidence base for the use of internal dosimetry in the clinical practice of molecular radiotherapy. EJNMMI. (2014) 41(10):1976–88. 10.1007/s00259-014-2824-5 24915892

[118] Aerts A Eberlein U Holm S Hustinx R Konijnenberg M Strigari L EANM Position paper on the role of radiobiology in nuclear medicine. EJNMMI. (2021) 48(11):3365–77. 10.1007/s00259-021-05345-9 PMC8440244 33912987

[119] Sgouros G Roeske JC McDevitt MR Palm S Allen BJ Fisher DR MIRD Pamphlet No. 22 (abridged): radiobiology and dosimetry of *α*-particle emitters for targeted radionuclide therapy. J Nucl Med. (2010) 51(2):311–28. 10.2967/jnumed.108.058651 20080889 PMC5680544

[120] Pinto MMP dL Santos NFG Amaral A. Current status of biodosimetry based on standard cytogenetic methods. Radiat Environ Biophys. (2010) 49(4):567–81. 10.1007/s00411-010-0311-3 20617329

[121] Allen B Huang C Clarke R. Targeted alpha anticancer therapies: update and future prospects. Biol: Targets Ther. (2014) 8:255–67. 10.2147/btt.s29947 PMC4232037 25422581

[122] International Commission on Radiological Protection. Radiological protection in medicine. ICRP publication 105. Ann ICRP. (2007) 37(6):1–63. Available at: https://www.icrp.org/publication.asp?id=ICRP%20Publication%20105 (Accessed April 06, 2023). 10.1016/j.icrp.2008.08.001 18762065

[123] Puerto S Marcos R Ramı´rez MJ Galofré Creus A Surrallés. Equal induction and persistence of chromosome aberrations involving chromosomes 1, 4 and 10 in thyroid cancer patients treated with radioactive iodine. Mutat Res Genet Toxicol Environ Mutagen. (2000) 469(1):147–58. 10.1016/S1383-5718(00)00064-4 10946251

[124] Violet D M’kacher R Adjadj E Dossou J de Vathaire F Parmentier C. Evidence of increased chromosomal abnormalities in French polynesian thyroid cancer patients. EJNMMI. (2005) 32:174–9. 10.1007/s00259-004-1662-2 15449047

[125] Garty G Bigelow AW Repin M Turner HC Bian D Balajee AS An automated imaging system for radiation biodosimetry. Microsc Res Tech. (2015) 78(7):587–98. 10.1002/jemt.22512 25939519 PMC4479970

[126] De Amicis A De Sanctis S Cristofaro SD Franchini V Regalbuto E Mammana G Dose estimation using dicentric chromosome assay and cytokinesis block micronucleus assay: comparison between manual and automated scoring in triage mode. Health Phys. (2014) 106(6):787–97. 10.1097/HP.0000000000000097 24776913

[127] Gruel G Grégoire E Lecas S Martin C Roch-Lefevre S Vaurijoux A Biological dosimetry by automated dicentric scoring in a simulated emergency. Radiat Res. (2013) 179(5):557–69. 10.1667/RR3196.1 23560627

[128] Beinke C Barnard S Boulay-Greene H De Amicis A De Sanctis S Herodin F Laboratory intercomparison of the dicentric chromosome analysis assay. Radiat Res. (2013) 180(2):129–37. 10.1667/RR3235.1 23862730

[129] Oestreicher U Samaga D Ainsbury E Antunes AC Baeyens A Barrios L RENEB Intercomparisons applying the conventional dicentric chromosome assay (DCA). Int J Radiat Biol. (2017) 93(1):20–9. 10.1080/09553002.2016.1233370 27766931

[130] M’Kacher R El Maalouf E Terzoudi G Ricoul M Heidingsfelder L Karachristou I Detection and automated scoring of dicentric chromosomes in nonstimulated lymphocyte prematurely condensed chromosomes after telomere and centromere staining. Int J Radiat Oncol Biol Phys. (2015) 91(3):640–9. 10.1016/j.ijrobp.2014.10.048 25596111

[131] Liu G. Revision of cytogenetic dosimetry in the IAEA manual 2011 based on data about radio-sensitivity and dose-rate findings contributing. FASEB J. (2022) 36(11):e22621. 10.1096/fj.202200769RR 36260291

[132] Wojda A Zietkiewics E Witt M. Effects of age and gender on micronucleus and chromosome nondisjunction frequencies in centenarians and younger subjects. Mutagenesis. (2007) 22(3):195–200. 10.1093/mutage/gem002 17284771

[133] Wilkins RC Romm H Kao TC Awa AA Yoshida MA Livingston GK Interlaboratory comparison of the dicentric chromosome assay for radiation biodosimetry in mass casualty events. Radiat Res. (2008) 169(5):551–60. 10.1667/RR1272.1 18439045

[134] Jaworska A Wojcik A Ainsbury EA Fattibene P Lindholm C Oestreicher U Guidance for using multibiodose tools in emergencies—for radiation emergency response Organisations in Europe. Printed by 07 Media, Oslo. Available at: https://www.reneb.net/wp-content/uploads/2017/09/multibiodose-guidance-small.pdf

